# Exploring the effects of cosolutes and crowding on the volumetric and kinetic profile of the conformational dynamics of a poly dA loop DNA hairpin: a single-molecule FRET study

**DOI:** 10.1093/nar/gky1122

**Published:** 2018-11-12

**Authors:** Satyajit Patra, Vitor Schuabb, Irena Kiesel, Jim-Marcel Knop, Rosario Oliva, Roland Winter

**Affiliations:** 1 Physical Chemistry I - Biophysical Chemistry, Faculty of Chemistry and Chemical Biology, TU Dortmund University, Otto-Hahn Street 4a, 44227 Dortmund, Germany; 2 Department of Chemical Sciences, University of Naples Federico II, Via Cinita, 80126 Naples, Italy

## Abstract

We investigated the volumetric and kinetic profile of the conformational landscape of a poly dA loop DNA hairpin (Hp) in the presence of salts, osmolytes and crowding media, mimicking the intracellular milieu, using single-molecule FRET methodology. Pressure modulation was applied to explore the volumetric and hydrational characteristics of the free-energy landscape of the DNA Hp, but also because pressure is a stress factor many organisms have to cope with, e.g. in the deep sea where pressures even up to the kbar level are encountered. Urea and pressure synergistically destabilize the closed conformation of the DNA Hp due to a lower molar partial volume in the unfolded state. Conversely, multivalent salts, trimethylamine-N-oxide and Ficoll strongly populate the closed state and counteract deteriorating effects of pressure. Complementary smFRET measurements under immobilized conditions at ambient pressure allowed us to dissect the equilibrium data in terms of folding and unfolding rate constants of the conformational transitions, leading to a deeper understanding of the stabilization mechanisms of the cosolutes. Our results show that the free-energy landscape of the DNA Hp is a rugged one, which is markedly affected by the ionic strength of the solution, by preferential interaction and exclusion of cosolvents as well as by pressure.

## INTRODUCTION

A significant portion of the global biosphere (∼62%) exists at a depth in excess of 1000 m and is therefore subject to high hydrostatic pressure (HHP) of 100 bar or more, and can reach up to 1 kbar in the Mariana Trench of the Pacific Ocean (1,2). A plethora of organisms are thriving under such extreme conditions, such as in the deep sea floor, marine hydrothermal vents and volcanic environments, which are also discussed to be the potential birthplace of life on Earth (3). Adaptation mechanisms must exist to stabilize biomolecular systems and ensure life under such harsh environmental conditions. Yancey et al. have found that extremophiles acquire a particular set of osmolytes to combat the extreme conditions and the amount of particular osmolytes such as trimethylamine-N-oxide (TMAO), increases with increasing depth of capture (4,5). To reveal the underlying mechanisms of adaptation, the combined effects of pressure and cosolutes mimicking intracellular conditions on the stability and conformational dynamics of biomolecular systems, such as membranes, proteins and nucleic acids, have to be explored (3). Apart from the biological relevance, high-pressure studies on biomolecular systems are also fundamental from the physical-chemical point of view (1,6–8). Whereas temperature dependent studies provide the change of enthalpy (Δ*H*) and entropy (Δ*S*) upon conformational transitions or reactions of biomolecules, complementary pressure studies provide volumetric information (Δ*V*), which are generally linked to changes in structure (packing) and hydration. According to Le Châtelier’s principle, pressure shifts the conformational equilibrium of the system towards states with smaller partial molar volume. The driving forces dictating pressure-induced conformational shifts are the release of packing defects or voids and changes in hydration (e.g. upon exposure of charged residues), resulting in an overall smaller volume of the system (1,8,9–17).

Even though HHP studies have become a common biophysical technique in studying the thermodynamics and kinetics of biomolecular systems, pressure studies on nucleic acids are still relatively scarce (9,12,14–21). In particular, the effect of cosolutes, such as salts, osmolytes and crowding agents mimicking intracellular conditions, on the folding landscape and structural dynamics of nucleic acids are still largely under research (17,18,21–24). Canonical DNA duplex structures such as B-DNA are generally found to be pressure resistant (14,15). Application of pressure decreases the Watson-Crick H-bond distance, favors base stacking interactions and hence stabilizes the double-stranded DNA structure (14,15). Conversely, only recently it has been found, pioneered by the Sugimoto, Chalikian and Macgegor labs, that noncanonical DNA structures, such as G-quadruplex DNA and polydA loop DNA hairpins, are rather sensitive to pressure (16,17,19–21).

**Figure 1.**
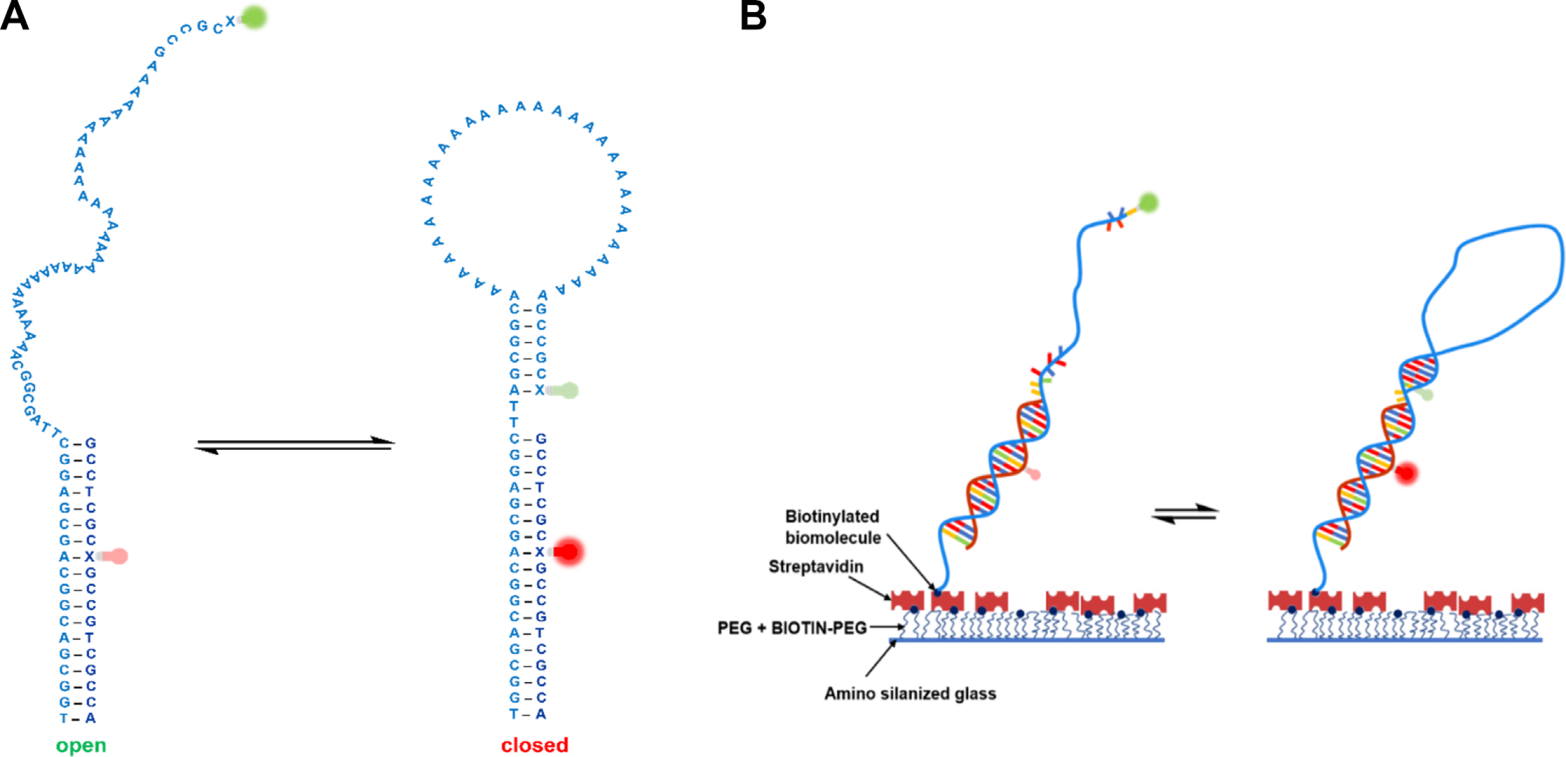
(**A**) Chemical structure of the open and closed conformational state of the Atto 550 (donor) and Atto 647N (acceptor) labeled DNA Hp. The positions of the donor (green) and acceptor (red) fluorophore are shown as fluorescent bulbs. The open state has a large donor-to-acceptor separation and therefore displays a low FRET. Loop formation in the closed state leads to a smaller donor-to-acceptor separation and therefore to a high FRET signal. Monitoring the FRET intensity versus time traces allows also to determine the dynamics (rate constants) of the conformational changes involved. (**B**) Schematic diagram of the surface preparation for the immobilization of the DNA Hp. First, the cover slip is coated with aminosilane and then PEGylated to avoid nonspecific binding of the biomolecule. In the next step, avidin is attached to the biotinylated surface. In the final step, biotin-labeled DNA Hp along with its fluorescent markers are attached to the avidinylated surface. The DNA Hp is labeled with biotin using a C6 spacer, which eliminates any detrimental effects of surface attachment and allows the molecule to undergo spontaneous conformational changes.

Herein, we have used a DNA hairpin (DNA Hp) that contains 32 adenine residues in the loop (Figure 1) (25), which has recently been found to be pressure sensitive (9,17). Hairpins regulate gene expression, act as target sites for protein recognition and nucleation sites for higher order RNA structures, and they play an important role in DNA recombination and transposition (26–28). Hairpins are also found in applications as biosensors and in DNA nanotechnology (29,30). The stem-loop hairpin structures are also very attractive systems from a biophysical point of view to investigate the base pairing and unpairing kinetics (25,31). The stability of these structures depends on a complex interplay of base stacking interactions, H-bonding and hydration (32). The solution environment plays an important role in modulating these interactions and controlling the structure, dynamics and function of these biomolecules (17,24,33).

Knowledge of the energy and conformational landscape of DNA hairpins is of fundamental biological interest. Despite a number of recent studies on DNA Hps, a deep understanding of the folding energy landscape of this class of biomolecules is still lacking and a subject of debate (17,31,34–37). The poly dA loop DNA hairpin was chosen owing to the significant pressure sensitivity compared to the double-stranded DNA and smaller loop DNA or RNA hairpins. Further, it is a well-characterized system under ambient buffer conditions. Initial studies, which also include fluorescence correlation spectroscopy (FCS), have revealed a two-state system with a distinct free energy barrier between them (31,34,35,38). Later studies based on single-molecule Förster resonance energy transfer (smFRET), diffusion decelerated FCS, dual-beam fluorescence cross correlation spectroscopy and laser temperature-jump studies have indicated a rather rugged energy landscape with possible involvement of stable and metastable intermediates, and recent smFRET studies on such hairpin systems again indicate a two-state behavior (17,36,39,40). In addition to the sketchiness of these data, the scenario of how different types of cosolutes affect the conformational landscape of such hairpin systems and the rate constants of conformational transitions remains still largely unexplored. Herein, we put a spotlight on the effect of various salts, osmolytes and crowding agents on the structural transitions of a DNA Hp (Figure 1A) by using smFRET, which has several advantages over ensemble techniques (41–44). FRET measures distance on the nanometer length scale (typically 2–10 nm) and therefore on the single-molecule level, which allows to distinguish different conformational states that are often masked in an ensemble experiment upon averaging. Further, the dynamics of the conformational transitions can be studied under equilibrium conditions, so synchronization is not necessary. On the other hand, pressure studies are carried out, which are perfectly suited in mapping volumetric (packing and hydrational) properties of the folding energy landscapes of the DNA Hp. Such smFRET measurements under high pressure have only recently been realized by utilizing a square-shaped silica microcapillary system, which provides minimum optical aberration and at the same time withstands pressures as high as 2 kbar (17).

We have chosen monovalent (K^+^), divalent (Mg^2+^) and trivalent (Co^3+^) cationic salts to investigate the effect of charge density on the folding energy landscapes of the DNA hairpin under pressure. Osmolytes are small, neutrally charged organic cosolutes accumulated by organisms to combat osmotic stress, such as high salinity, HHP and the presence of denaturants. We used two commonly used osmolytes, TMAO as a stabilizing and urea as a destabilizing osmolyte. Ficoll has been used to study the effect of macromolecular crowding on the folding energy landscapes of the DNA Hp. Ficoll is a copolymer of sucrose and epichlorohydrin and forms a network structure at higher concentrations (45). All pressure dependent smFRET measurements were carried out under freely diffusing condition. The volume changes (Δ*V*) obtained for the conformational transitions of the DNA hairpin in the absence and presence of the cosolutes from the high-pressure smFRET measurements provide a novel mechanistic understanding of packing and hydration effects in dictating the conformational preferences. Additional smFRET experiments carried out under immobilized conditions at ambient pressure revealed the underlying dynamic information (rate constants) of the conformational transitions, which is inaccessible from diffusion-based measurements as the observation time is limited by diffusion. Moreover, smFRET under immobilized measurements allowed us to unambiguously identify the presence of low-populated and intermediate states in the course of conformational transitions, which are difficult to assign from diffusion-based measurements, only. Overall, the present study provides crucial volumetric and kinetic information on the DNA Hp in the presence of biologically relevant cosolutes. Moreover, the results obtained are also immensely helpful in understanding the adaptation mechanisms of extremophiles used to combat environmental stresses.

## MATERIALS AND METHODS

### DNA sequence and sample preparation

The nucleotide sequence for the hairpin strands used in the smFRET measurements under freely diffusing and immobilized conditions are H1: 5′-biotin-TGG CGA CGG CAG CGA GGC TTA GCG GCA AAA AAA AAA AAA AAA AAA AAA AAA AAA AAA AGC CGC X-3′ (X is T-Atto 550) and H2: 5′-TGG CGA CGG CAG CGA GGC TTA GCG GCA AAA AAA AAA AAA AAA AAA AAA AAA AAA AAA AGC CGC X-3′ (X is T-Atto 550), respectively. The sequence of the complementary strand is A2: 5′-GCC TCG CXG CCG TCG CCA-3′ (X is T-Atto 647 N). The oligonucleotide strands were synthesized and fluorescently labeled by IBA Life Solutions GmbH (Göttingen, Germany). The complementary oligonucleotide strands (H1 and A2, H2 and A2) were annealed according to a reported protocol to prepare the double-stranded DNA hairpin. Both the hairpin strand (H1 or H2) and the complementary strand (A2) were mixed in a 1:1 ratio in buffer containing 20 mM Tris–HCl, 50 mM NaCl (pH 8.0) to a final concentration of 1 pmol μL^−1^. The annealing was performed by first heating the sample at 95°C for 5 min, followed by a gradual cooling to room temperature at a rate of −0.5°C min^−1^ using a thermocycler.

TMAO, urea, Ficoll PM 70, tris(hydroxymethyl)aminomethane hydrochloride (Tris–HCl), molecular biology grade potassium chloride (KCl) ≥ 99.0%, magnesium chloride (MgCl_2_) anhydrous (≥ 98.0%) and hexammine Co(III) chloride ([Co(NH_3_)_6_]Cl_3_) were procured from Sigma-Aldrich (Germany) and used as received. D-glucose, glucose oxidase from *Aspergillus niger*, catalase from bovine liver were purchased from Sigma-Aldrich (Germany), 6-hydroxy-2,5,7,8-tetramethylchromane-2-carboxylic acid (Trolox) from Fisher Scientific GmbH (Germany). Sodium chloride (NaCl), DNase, Ribonuclease free tips were purchased from VWR International GmbH (Germany). The high-pressure smFRET measurements were carried out in a square-shaped flexible fused silica capillary (WWP 050375, Polymicro Technologies), which was procured from CM Scientific (UK). Bottom-less flow channels (sticky slide VI^0.4^) and glass coverslips with geometry 25 mm × 75 mm and thickness 175 μm were purchased from Ibidi GmbH (Martinsried, Germany) to prepare the sample chamber with 6 channels for the smFRET measurement under immobilized conditions. N-[3-(trimethoxysilyl)propyl]ethylenediamine for the aminosilanization of the coverslip in immobilized smFRET measurements was purchased from abcr GmbH (Karlsruhe, Germany). Methoxy polyethylene glycol-succinimidyl carbonate (mPEG-SC) and biotinylated polyethylene glycol-succinimidyl carbonate (Biotin-PEG-SC) for the PEGylation of the aminosilanized coverslip were obtained from Laysan Bio Inc. (USA). Streptavidin from *Streptomyces Avidini* was procured from Sigma-Aldrich (Germany). Sodium bicarbonate (NaHCO_3_) was purchased from Biochrom GmbH (Berlin, Germany). All the measurements were performed in a buffer containing 20 mM Tris–HCl, 15 mM NaCl (pH 7.5). The buffer also contains 3 wt% D-glucose, 0.02 mg mL^−1^ catalase, 0.1 mg mL^−1^ glucose oxidase and 1 mM trolox to reduce photobleaching and photoblinking of the fluorophores. The buffer solutions were filtered using Whatman puradisc 30 syringe filters (GE Healthcare Life Sciences) of pore size 0.2 μm to remove any large particles from the solution.

### High-pressure methodology and smFRET measurements

High-pressure smFRET measurements under freely diffusing conditions were carried out in a square shaped flexible fused silica capillary with outer diameter 360 μm and inner diameter 50 μm (17,46). The capillary serves both as an optical window and mechanical body of the high-pressure cell. The optical window thickness of the capillary is ∼150 μm resembling the standard coverslip used in smFRET measurements. Details of this set up are fully described in a recent report (9). The smFRET measurements were carried out in a commercial time resolved confocal fluorescence microscope (MicroTime 200, PicoQuant). Details of the setup are described elsewhere (47). The pulsed interleaved excitation (PIE) FRET technique was used to filter the signal of the dually labeled species from the singly labeled species. In PIE-FRET, alternating laser excitation is used such that the fluorescence due to donor excitation appears in the early time window and fluorescence due to direct excitation of the acceptor appears in the late time window (48–50). Such information is used to determine the photon stoichiometry, which allows one to separate the dually labeled species from either donor only labeled or acceptor only labeled species. The fluorescence coming from dually labeled species only are used to calculate the FRET efficiency (*E*). Donor and acceptor fluorophores are excited by using pulsed diode lasers (LDH series, PicoQuant) with pulsed repetition rate of 20 MHz of wavelength 560 and 635 nm, respectively. The excitation power in the diffusion-based smFRET mode was 12 μW, while for the immobilized-based smFRET measurements, an excitation power of 0.7 μW was used. The excitation light of the green and red laser is reflected to the entrance port of the microscope with the help of a quad band dichroic mirror (ZT 405/488/560/640, Chroma). The fluorescence was collected in the same optical path guided through a dichroic mirror, a 575 nm long pass filter (HQ 575 LP, Chroma), and spatially filtered by using a 50 μm pinhole, which only allowed the light to pass which comes from the focal spot, and thus defined the observation volume. The fluorescence light coming from the donor and acceptor fluorophore were then spectrally separated into two detection channels by using a dichroic mirror (FF 650 Di 01, Semrock). In addition to that, two band pass filters FF01–593/40 and FF01–676/29 were used in front of the donor and acceptor fluorescence detection channel, respectively, to further suppress spectra leakage and background signals. Two SPCM-AQR (Perkin Elmer Inc.) series single photon avalanche diodes (SPAD) were used to detect the donor and acceptor fluorescence. A time correlated single photon counting unit with the TimeHarp 200 PCI (Peripheral Component Interconnect) board in a Time-Tagged-Time Resolved mode was used to store each photon with individual timing and channel information to generate the donor and acceptor fluorescence traces with time.

### Analysis of smFRET data under freely diffusing conditions

All the high-pressure smFRET measurements were carried out in solution under freely diffusing conditions. Each transit of the donor and acceptor fluorophore labeled DNA hairpin through the observation volume gives rise to characteristic fluorescence signals, also known as fluorescence bursts, due to multiple excitation and emission cycles. These fluorescence bursts were recorded photon by photon in the donor and acceptor fluorescence detection channels and were binned into a 1 ms time window, which is close to the diffusion time of the DNA hairpin, to generate the characteristic fluorescence intensity versus time traces in the donor and acceptor channel. The concentration of the DNA hairpin was kept at ∼200–300 pM to ensure that on average only one molecule is present in the confocal volume at a given time. A threshold criterion was used to separate the single-molecule fluorescence bursts from background noise. Only when the sum of the fluorescence photons in a time bin (1 ms) is larger than a threshold value, it is regarded as a single-molecule event (51,52). The photons above the threshold value in each burst were used to calculate the FRET efficiency, defined by *E* = *n*_A_/(*n*_A_+*γ* *n*_D_), where *n*_A_ and *n*_D_ are the number of photons in the acceptor and donor channel, respectively; *γ* is a correction factor that accounts for the differences in the fluorescence quantum yield (*ϕ*_A_ and *ϕ*_D_) and detection efficiency (*η*_A_ and *η*_D_) of the donor and acceptor fluorophore, respectively, defined as *γ* = (*ϕ*_A_*η*_A_)/(*ϕ*_D_*η*_D_); *γ* = 0.88 for the donor-acceptor pair Atto 550 and Atto 647N and the SPCM AQR series SPAD detectors.

### Measurement of smFRET and data analysis under immobilized conditions

To study the conformational dynamics over an extended period of time, the molecule needs to be localized in space. This is achieved by immobilizing the molecule on the glass coverslip via biotin–streptavidin interaction using reported procedures (44,53). The important point in the surface immobilization of biomolecules is to avoid nonspecific binding of the molecule on the glass surface. In the very first step, the glass coverslip is cleaned using a plasma cleaner to remove any organic impurities, which is followed by washing with MilliQ water and drying in a nitrogen stream. Surface passivation is initiated with aminosilanization of the glass coverslip. To this end, the glass coverslip is dipped into a solution of 1% (v/v) 3-aminopropyltriethoxysilane in slightly acidic methanol (5% v/v acetic acid) for 10 min. Excess silane is washed away with MilliQ water and blow drying. The silane coated coverslips are then fixed to the sticky slide to prepare the flow channel. Next, the flow channels are incubated with a solution of 98% (w/v) mPEG-SC and 2% (w/v) BIOTIN-PEG-SC in 100 mM NaHCO_3_ buffer (pH 8.25) for 3–4 h to prepare the biotinylated glass surface. The flow channels are then repeatedly rinsed with MilliQ water to remove any excess unattached PEG, which is followed by drying in a nitrogen gas stream. In the next step, the glass surface is avidinylated by incubating the flow channel with a 1 mg mL^−1^ solution of streptavidin in a buffer containing 20 mM Tris–HCl, 15 mM NaCl, pH 7.5, for 15 min, and the excess unattached streptavidin is washed away by rinsing with buffer multiple times. Finally, the biotin-labeled DNA hairpin with fluorescent markers is attached to the avidinylated glass surface by incubating the flow channel with a 20 pM solution of the DNA hairpin in buffer for 20 min. The unattached DNA hairpins are removed by repeated rinsing with buffer solution. A scheme of the immobilization setup is depicted in Figure 1B. Before starting the measurements, the flow channel is filled with imaging buffer containing 3 wt% D-glucose, 0.02 mg mL^−1^ catalase, 0.1 mg mL^−1^ glucose oxidase and 1 mM trolox to reduce the photoblinking and photobleaching of the fluorophores (54,55). The corresponding cosolvents and crowding agent were added to the imaging buffer prior to the measurements.

At a 20 pM concentration, around 50 single molecules are immobilized in an area of 12 × 12 μm^2^. The bright spots indicating single molecules remain well separated. Point measurements were taken on these bright spots. Single-step photobleaching of the donor and acceptor fluorescence recorded indicate that the FRET is due to a single molecule. The donor and acceptor fluorescence intensity versus time trajectories from about 30 single molecules were exported as a .dat file for further analysis in ebFRET, which is a freely available programme run in Matlab to obtain the kinetic information of the transitions between different conformational states (56,57). FRET efficiency (*E*) versus time trajectories were obtained from the donor (Atto 550) and acceptor (Atto 647N) intensity versus time series. *E* is calculated by dividing the donor intensity by the sum of the donor and acceptor intensity at each point in the trajectories (see the ESI). Each FRET state (*E-*value) represents a distinct conformational state. By determining the time, the molecule stays in one particular FRET state (denoted as dwell time) before making a transition to another state, provides the kinetics for that conformational transition. The dwell time for each transition in the *E* versus time trajectories are determined using the Hidden Markov Model (HMM) in the ebFRET software package. HMM allows to identify the states and the probability of transitions between different states in an otherwise noisy time series using a variational Bayesian approach (58). The raw dwell time distribution histograms obtained are then integrated to generate a cumulative plot where each point indicates the number of counted events that have a dwell time less than or equal to the specified time (59,60). These cumulative distribution plots are then fitted with single exponential functions to obtain the dwell time of that particular FRET state, which in turn provides the rate constant (*k*) for that transition.

### Ensemble FRET measurements

Ensemble FRET measurements were carried out in a K2 multifrequency phase and modulation fluorimeter (ISS Inc. USA). The concentration of the donor (Atto 550) and acceptor (Atto 647N) fluorophore labeled DNA Hp was 0.17 μM. The same Tris buffer (20 mM Tris–HCl, 15 mM NaCl, pH 7.5) was used also in the ensemble FRET measurements. The excitation wavelength (*λ*_ex_) was 550 nm, which corresponds to the absorption maximum of the Atto 550 fluorophore.

## RESULTS

### Volumetric profile of the unfolding process of the DNA Hp in the presence of salts

**Figure 2.**
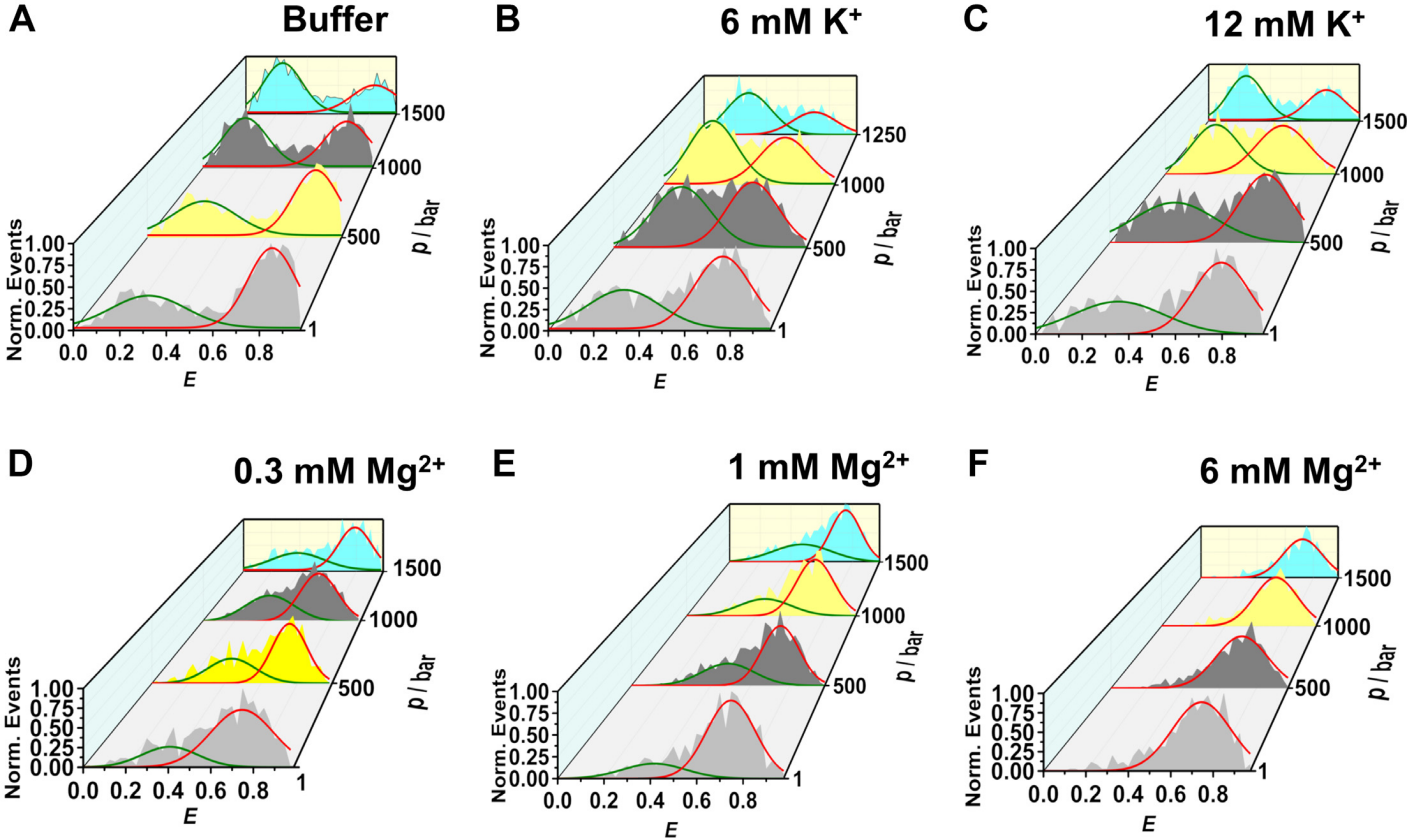
Normalized 3D plots of the FRET efficiency (*E*) histograms of the DNA Hp in (**A**) neat buffer (20 mM Tris–HCl, 15 mM NaCl, pH 7.5), in the presence of (**B**) 6 mM K^+^, (**C**) 12 mM K^+^, (**D**) 0.3 mM Mg^2+^, (**E**) 1 mM Mg^2+^ and (**F**) 6 mM Mg^2+^ at different pressures, *p*. The green and red curves represent Gaussian fits to the *E*-histograms representing open and closed conformations, respectively.

High-pressure smFRET measurements under freely diffusing conditions were carried out in the presence of monovalent (K^+^), divalent (Mg^2+^) and trivalent cationic (Co^3+^) salts to quantify the effect of charge density on the volumetric profile of the conformational transitions of the DNA Hp. This is of particular importance as the ionic strength affects the hydration and packing arrangement of the DNA Hp, which dictate its conformational stability in the intracellular environment. Figure 2 exhibits the FRET efficiency (*E*) histograms of the DNA Hp in the presence of different concentrations of K^+^ and Mg^2+^. The *E*-histograms corresponding to 0.3, 1 and 6 mM Co^3+^ are shown in Supplementary Figure S1 (see the ESI). The two peaks seen in the *E*-histograms clearly reveal two conformations. The low-FRET peak at *E* ≈ 0.3 represents the open (unfolded) conformation where the donor-to-acceptor distance is large. The high-FRET peak at *E* ≈ 0.80 represents the closed (folded) conformation where the donor-to-acceptor distance is short. Pressure application in neat buffer solution gradually populates the low-FRET species (*E* ≈ 0.3), i.e. leads to unfolding of the DNA Hp (Figure 2A). Addition of 6 and 12 mM K^+^ does not have any significant effect on the conformational stability of the DNA Hp against pressure. Conversely, the addition of Mg^2+^ and Co^3+^ strongly stabilizes the high-FRET species (closed state) and counteracts the destabilization effect of pressure such that in 6 mM Mg^2+^ or Co^3+^ the closed state prevails (Figure 2 and Supplementary Figure S1).

**Figure 3.**
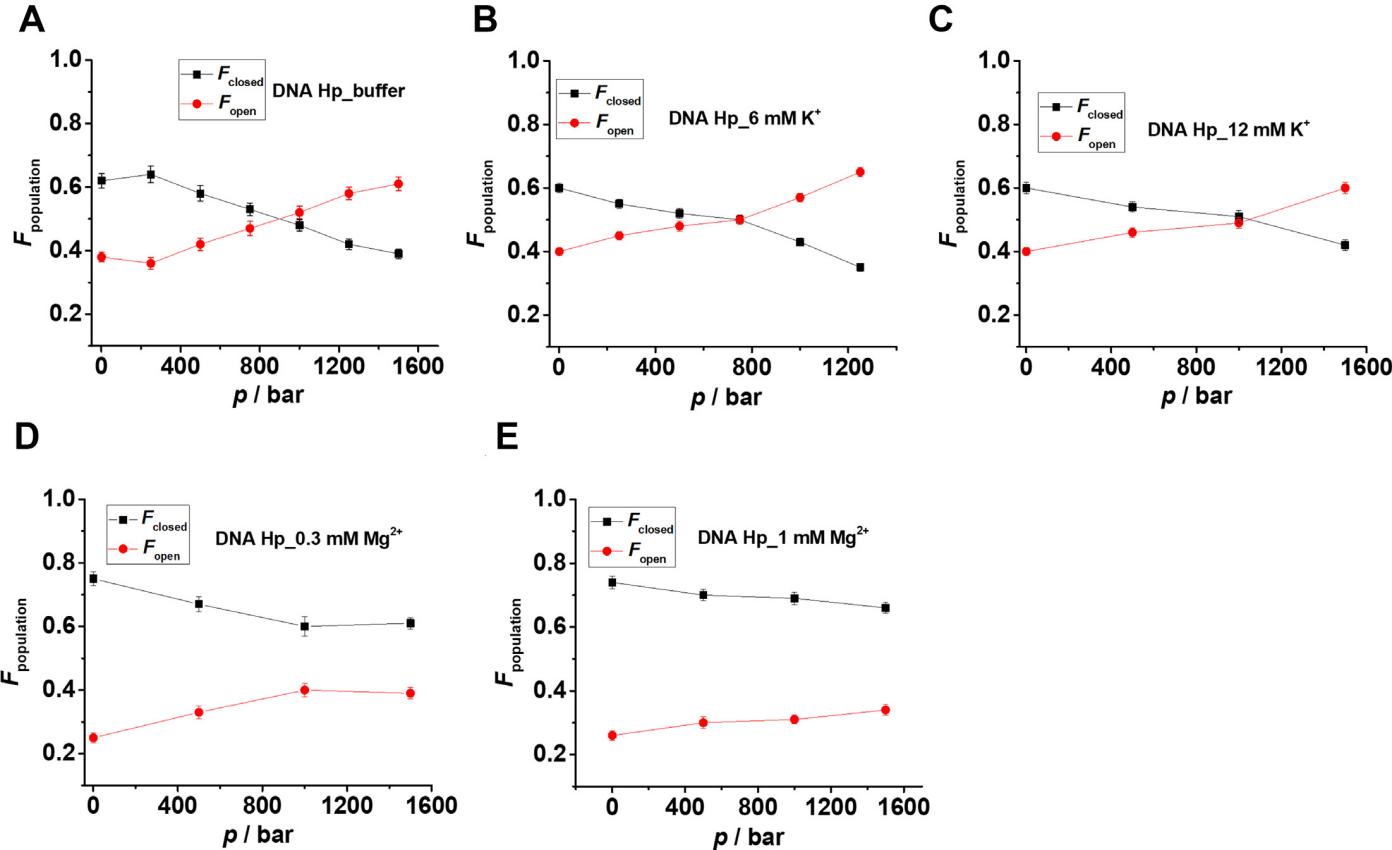
Fraction (*F*) of closed (black square, *E* ≈ 0.7–0.8) and open (red circle, *E* ≈ 0.3–0.4) conformational states as determined from the peak areas of the Gaussian fits in Figure 2 at different pressures for (**A**) neat buffer, (**B**) 6 mM K^+^, (**C**) 12 mM K^+^, (**D**) 0.3 mM Mg^2+^and (**E**) 1 mM Mg^2+^. The equilibrium constant (*K*_eq_) for the closed to open state transition is determined from *K*_eq_ = *F*_open_/*F*_closed_ at different pressures (*T* = 25°C) in neat buffer, 6 mM K^+^, 12 mM K^+^, 0.3 mM Mg^2+^ and 1 mM Mg^2+^. From the slope of the ln*K*_eq_ versus *p* plot, the transition volume (Δ*V*^o^) for the closed to open state conformational transition is determined as (dln*K*_eq_/d*p*)*_T_* = −Δ*V*^o^/(*RT*) (see Supplementary Figure S2).

The population of different conformational states is determined from the peak areas of the Gaussian fits representing the different conformations. *F*_open_ = (area of the low-FRET peak)/total area, represents the fraction of the open state, and *F*_closed_ = 1 - *F*_open_ the fraction of closed (high-FRET peak) state. Figure 3 and Supplementary Figure S1 show *F*_open_ and *F*_closed_ as a function of pressure in the absence and presence of the salts. From the plots it is quite evident that the addition of Mg^2+^ and Co^3+^ strongly stabilizes the high-FRET species (closed) with *E* ≈ 0.8 and also counteracts the destabilization effect of pressure, while the effect of Na^+^ is negligible on the stability of the DNA Hp. The equilibrium constant (*K*_eq_) for the closed-to-open transition, i.e. for unfolding of the DNA Hp, is determined from the *F*_closed_ and *F*_open_ data at different pressures, both in absence and presence of salts (Supplementary Figures S1 and S2). From the slope of the plot ln*K*_eq_ versus *p*, the transition volume Δ*V*^o^ = *V*^o^_open_ - *V*^o^_closed_ for unfolding is determined. The data are listed in Table 1. The Δ*V*^o^-value in neat buffer is found to be about −18 cm^3^mol^−1^, in good agreement with literature data (9). A negative volume change associated with unfolding means that the open conformation has a smaller partial molar volume than the closed state. Upon increasing the salt concentrations, particularly in the presence of Mg^2+^ and Co^3+^, Δ*V*^o^ changes from −17.7 cm^3^mol^−1^ in neat buffer to −5.9 and −5.2 cm^3^mol^−1^ in the presence of 1 mM Mg^2+^ and 1 mM Co^3+^, respectively (Table 1). The smaller volume differences account for the strong counteracting effects of the salts on the pressure-induced destabilization of the closed state. The stability of the DNA Hp follows the order Co^3+^ > Mg^2+^ > K^+^, i.e. increases with increasing charge density of the salt. Complementary ensemble fluorescence measurements carried out in the presence of various salt concentrations support these observations found in single-molecule measurements (Supplementary Figures S3 and S4).

**Table 1.** Transition volume (Δ*V*^o^) for unfolding of the DNA Hp obtained from the slope of ln*K*_eq_ versus *p* plot (Supplementary Figures S1, S2 and S5) in neat buffer and in the presence of different concentrations of cosolutes (*T* = 25°C)

[Salt]	Δ*V*^o^ / cm^3^mol^−1^
Neat buffer	−17.7 ± 1.7
6 mM K^+^	−18.8 ± 2.2
12 mM K^+^	−12.7 ± 2.2
0.3 mM Mg^2+^	−11.1 ± 3.5
1 mM Mg^2+^	−5.9 ± 1.0
0.3 mM Co^3+^	−7.9 ± 1.9
1 mM Co^3+^	−5.2 ± 2.9
1 M TMAO	−7.8 ± 0.9
1 M urea	−29.5 ± 3.8
20 wt% Ficoll	−6.6 ± 1.9

### Volumetric profile of the unfolding process of the DNA Hp in the presence of osmolytes and crowders

**Figure 4.**
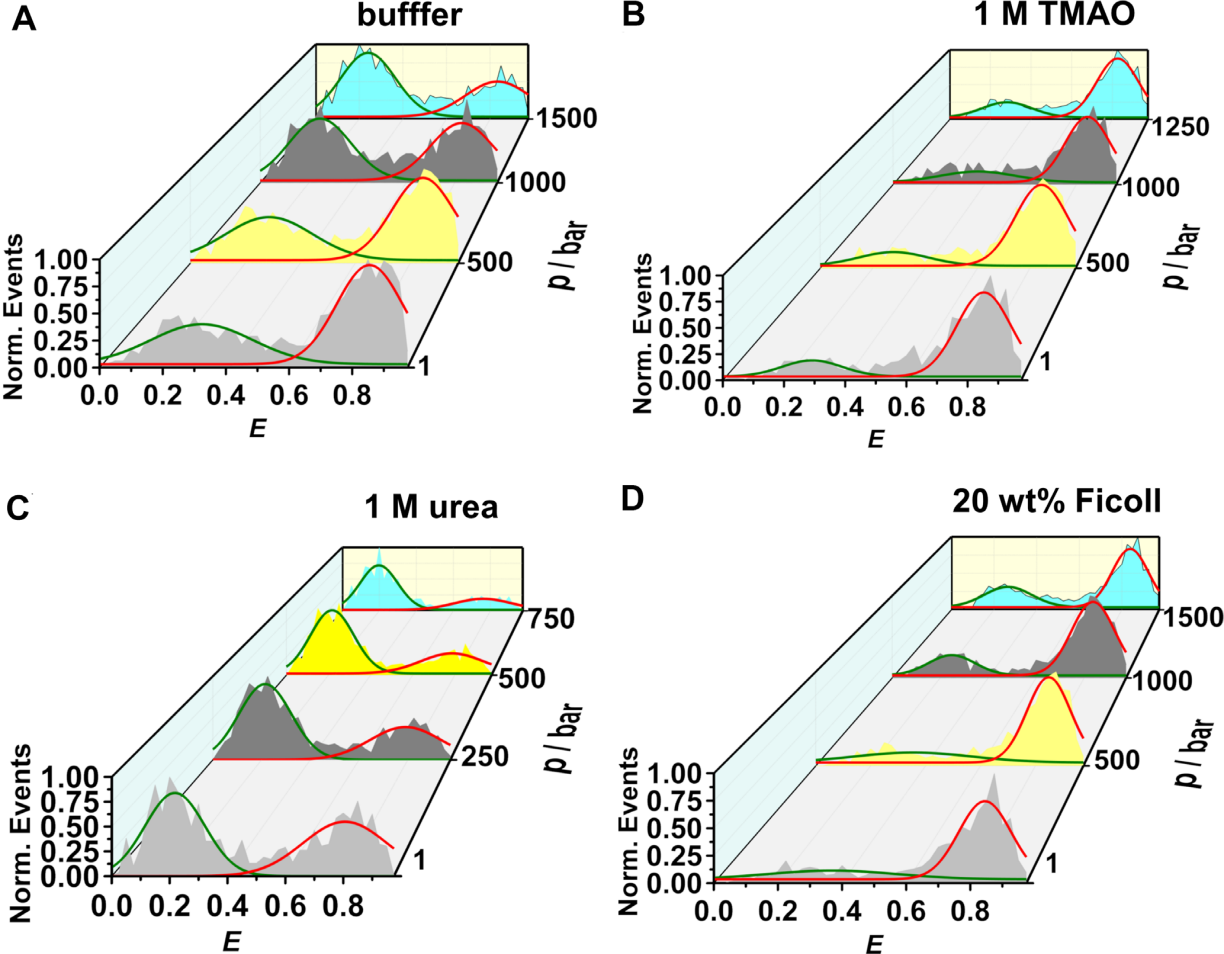
Normalized FRET efficiency (*E*) histograms of the DNA Hp in (**A**) neat buffer, in the presence of (**B**) 1 M TMAO, (**C**) 1 M urea and (**D**) 20 wt% Ficoll, respectively. TMAO and Ficoll stabilize the high FRET species (closed state), thereby counteracting the destabilization effect of pressure.

High-pressure smFRET measurements were also carried out in the presence of osmolytes and crowders. This is of particular physiological relevance as the cell takes up osmolytes to cope with environmental stresses and the cellular interior is also crowded with macromolecules (61–64). Herein, we have used examples of most common stabilizing and destabilizing osmolytes, TMAO and urea, respectively, and the polysaccharide Ficoll to mimic intracellular crowding conditions. The *E*-histograms obtained in neat buffer and in the presence of 1 M TMAO, 1 M urea and 20 wt% Ficoll are shown in Figure 4. It is clearly seen that TMAO and 20 wt% Ficoll stabilize the closed state (*E* ≈ 0.8). On the other hand, 1 M urea destabilizes the native fold of the DNA Hp, and, along with pressure synergistically populates the open conformation of the DNA Hp.

**Figure 5.**
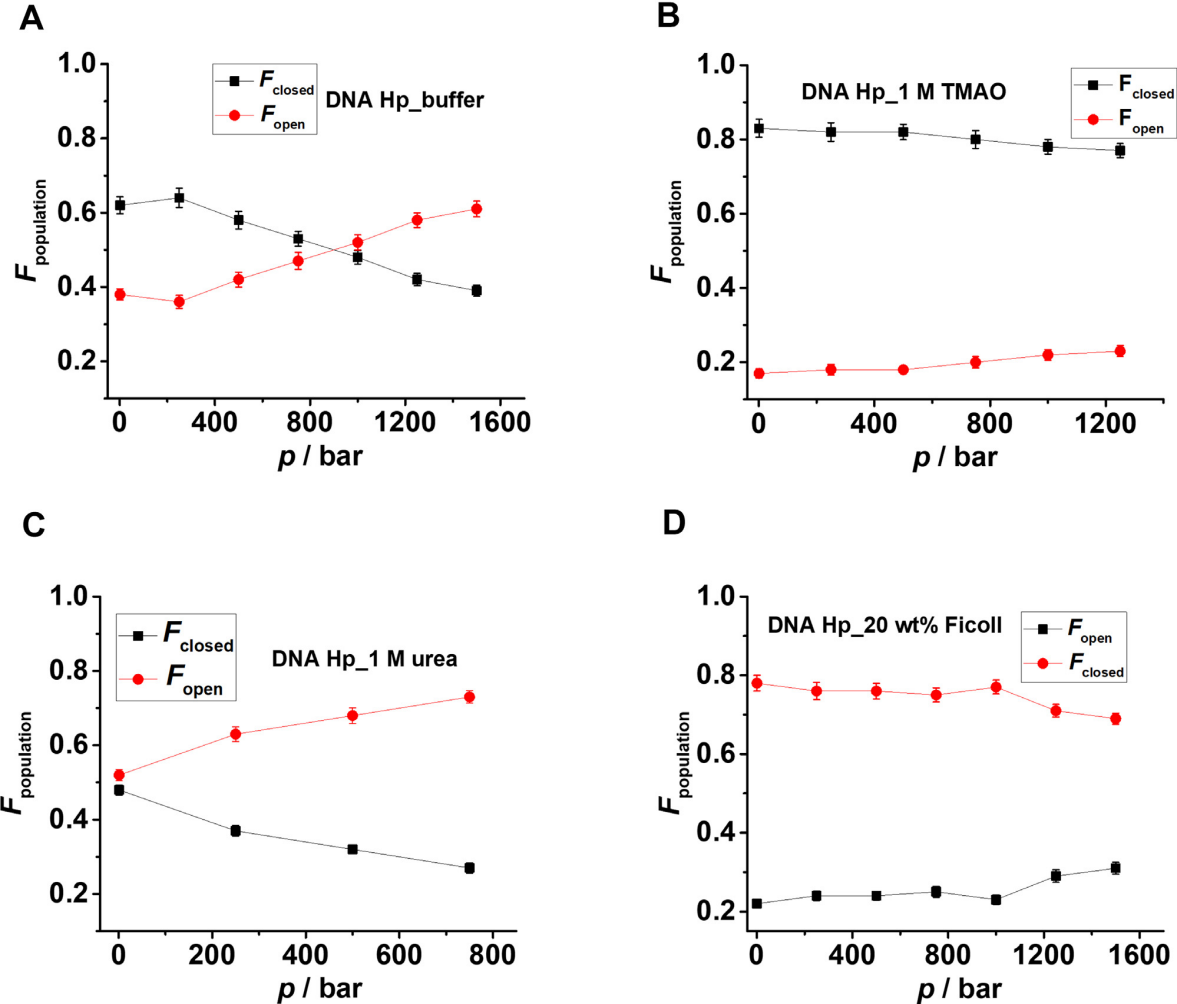
Plot of fraction of the open and closed conformations *F*_open_, *F*_closed_ of the DNA Hp as a function of pressure in (**A**) neat buffer without any cosolvent, in the presence of (**B**) 1 M TMAO, (**C**) 1 M urea and (**D**) 20 wt% Ficoll. *F*_open_ and *F*_closed_ are determined from the peak areas of the Gaussian fits in Figure 4. Equilibrium constants (*K*_eq_) for unfolding, defined as *K*_eq_ = *F*_open_/*F*_closed_, are determined and plotted as ln*K*_eq_ versus *p* in neat buffer, 1 M TMAO, 1 M urea and 20 wt% Ficoll in Supplementary Figure S5. From the slope of the plots, the transition volumes for unfolding are determined (see Supplementary Figure S5).

The population of the different conformational states is determined from the peak areas of the Gaussian fits shown in Figure 5. 1 M TMAO and 20 wt% Ficoll stabilize the closed state and fully compensate the deteriorating effect of pressure up to the whole pressure range covered and found in the biotic window (∼1000 bar). *K*_eq_ is plotted as a function of pressure both in neat buffer and in the presence of osmolytes and crowders in Supplementary Figure S5, the Δ*V*^o^ data are also presented in Table 1. The Δ*V*^o^ decreases from −17.7 cm^3^mol^−1^ in neat buffer to −7.8 and −6.6 cm^3^mol^−1^ in the presence of 1 M TMAO and 20 wt % Ficoll, respectively, thereby rendering the unfolded state less favorable upon compression. Ensemble fluorescence measurements carried out in the presence of 20 wt% Ficoll support these findings (Supplementary Figure S6). On the other hand, in the presence of 1 M urea, Δ*V*^o^ amounts to −29.5 cm^3^mol^−1^, which is considerably larger than the Δ*V*^o^ obtained for neat buffer. As a result, urea assists in pressure-induced destabilization of the folded state.

### smFRET measurements under immobilized conditions

**Figure 6.**
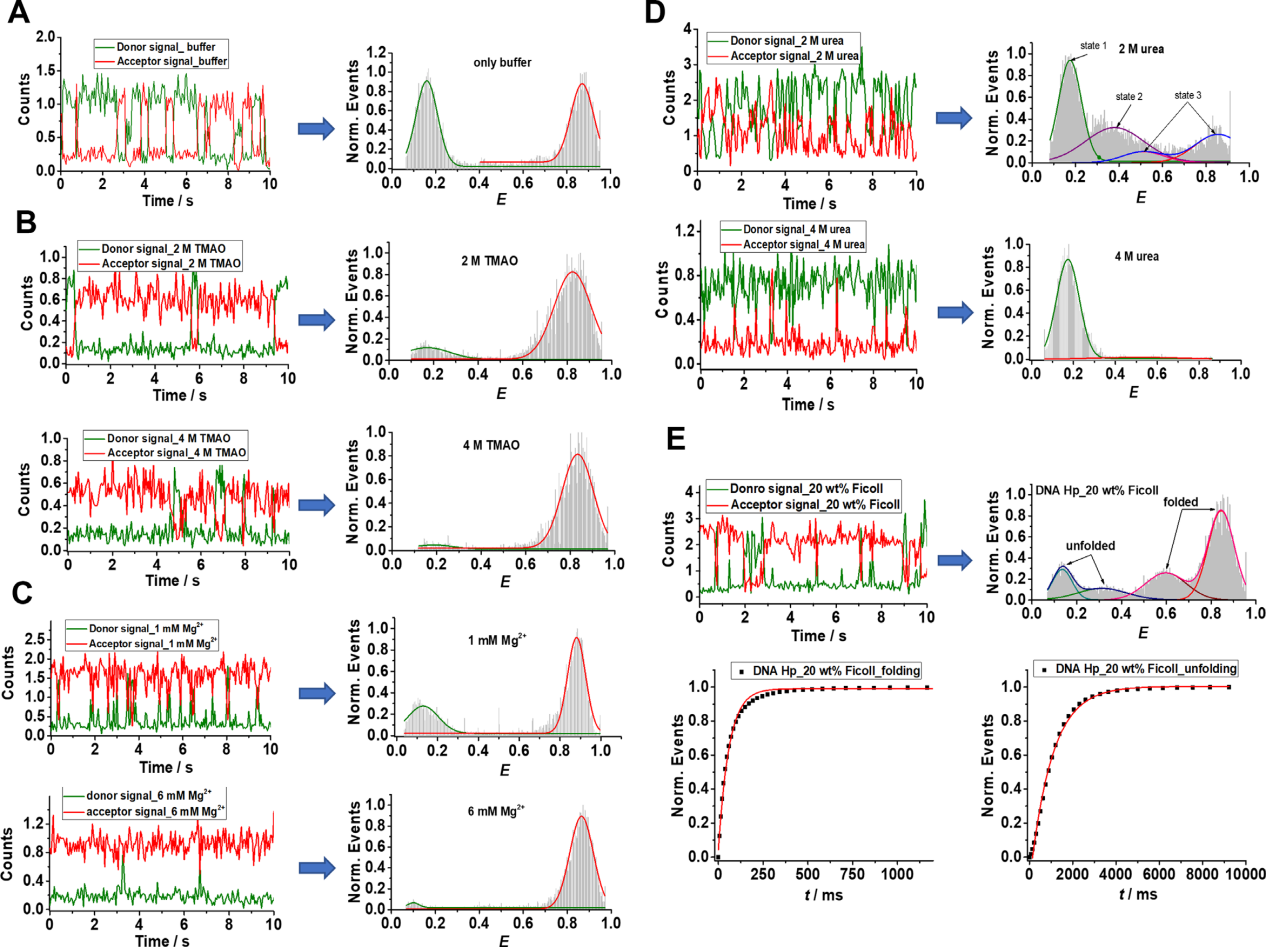
Donor (green) and acceptor (red) intensity versus time traces obtained for a typical single Atto 550 and Atto 647N labeled DNA Hp in (**A**) neat buffer and in the presence of (**B**) TMAO, (**C**) Mg^2+^, (**D**) urea and (**E**) 20 wt% Ficoll. The FRET efficiency (*E*) is calculated at each point of these traces using *E* = *n*_A_/(*n*_A_+*γ n*_D_), where *n*_A_ and *n*_D_ are the counts in the donor and acceptor channel, respectively, and *γ* is the correction factor for detector efficiency, to obtain the FRET time series as shown in Supplementary Figure S7. The FRET efficiency (*E*) histograms are obtained from the HMM fits to the FRET time series of about 30 single molecules. *E*-histograms are fitted to a bimodal Gaussian distribution in case of neat buffer, TMAO, Mg^2+^ and 4 M urea, where each Gaussian curve represents a distinct conformational state. In case of 2 M urea, three conformational states were identified from the FRET time series (see Supplementary Figure S11), where state 1 has a FRET distribution (green curve) centered at *E* = 0.17 ± 0.01, state 2 (purple color) at *E* = 0.38 ± 0.01 and state 3 displays a bimodal FRET distribution (blue color overall fit) at *E* = 0.51 ± 0.01 (pink color) and *E* = 0.86 ± 0.01 (red color). In case of 20 wt% Ficoll, the FRET-time series (see Supplementary Figure S14) display a two-state transition with folded and unfolded conformations, each state displaying a bimodal FRET distribution (blue and pink overall fit) centered at *E* = 0.60 ± 0.01/*E* = 0.85 ± 0.01 and *E* = 0.13 ± 0.01/*E* = 0.31 ± 0.01, respectively. The folding and unfolding kinetic constants are determined from the exponential fits to the cumulative dwell time distribution analysis, which is derived by integrating the raw dwell time distribution histograms as shown in Supplementary Figure S7. The raw dwell time distribution is directly obtained from the HMM fits to the FRET time series (see Supplementary Figure S7). Characteristic cumulative dwell time distribution plots for folding and unfolding are shown for the case of 20 wt% Ficoll (e).

The smFRET measurements were also carried on surface-attached DNA Hp to observe the opening and closing dynamics directly over longer times scales and to determine the underlying kinetic constants. Owing to the design and dimensions of the high-pressure capillary, such measurements can be carried out under ambient conditions, only. Representative fluorescence intensity traces of a single Atto 550 and Atto 647N labeled DNA Hp in neat buffer and in the presence of additives are shown in Figure 6. The *E-*histograms are obtained from at least 30 of such single-molecule fluorescence traces. The opening and closing dynamics of the DNA Hp produces anticorrelated donor and acceptor fluorescence, a typical FRET signature. In neat buffer, the DNA Hp spends roughly equal time in the open and closed state as can be seen from the fluorescence trace and the *E*-histogram (Figure 6A). With increasing TMAO concentration, the DNA Hp resides increasingly longer in the closed state as indicated by the predominant acceptor (red) fluorescence in the time trace and the increasing events with high *E-*value (∼0.85) in the *E*-histograms (Figure 6B). A similar behavior is observed in case of Mg^2+^ (Figure 6C). The *E*-histograms reveal a two-state transitional behavior for the DNA Hp in neat buffer, in the presence of Mg^2+^ as well as in TMAO solution (in 90% of the cases for 2 M TMAO).

The dynamic constants between the open and closed states are obtained from the Hidden Markov Model (HMM) fit to the FRET time series (*E* versus time) as shown exemplarily in Supplementary Figure S7, which yields the dwell time, i.e. the time the molecule spent in one FRET state before transitioning to another one. The opening (unfolding) and closing (folding) rate constants are determined from single exponential fits to the cumulative dwell time histograms of the closed and open state, respectively (Supplementary Figure S8) and are summarized in Table 2.

**Table 2.** Folding and unfolding rate constants of the DNA Hp in neat buffer and in the presence of TMAO, Mg^2+^, 4 M urea and Ficoll, determined from single exponential fits to the dwell time distribution histograms of the open and closed state as shown in Supplementary Figures S8, S13, 6

Solvent	${k_{\rm{f}}}/\ {{\rm{s}}^{ - 1}}$	${k_{\rm{u}}}/\ {{\rm{s}}^{ - 1}}$	$k_{{\rm{f\ }}}^{{\rm{corr}}}/\ {{\rm{s}}^{ - 1}}$	$k_{{\rm{u\ }}}^{{\rm{corr}}}/\ {{\rm{s}}^{ - 1}}$
Neat buffer	7.5 ± 0.2	3.13 ± 0.05		
2 M TMAO	5.7 ± 0.2	1.29 ± 0.04	10.5 ± 0.4	2.39 ± 0.07
4 M TMAO	5.9 ± 0.1	1.25 ± 0.05	23.2 ± 0.4	4.01 ± 0.16
1 mM Mg^2+^	11.9 ± 0.3	1.25 ± 0.02		
6 mM Mg^2+^	13.3 ± 0.2	1.65 ± 0.04		
4 M urea	1.94 ± 0.03	14.5 ± 0.5		
20 wt % Ficoll	11.7 ± 0.4	1.00 ± 0.02	48.9 ± 0.1	4.13 ± 0.08

In case of TMAO and 20 wt% Ficoll, the rate constants had to be corrected (index corr) for changes in viscosity (1.65 and 3.50 mPa⋅s of 2 M and 4 M TMAO, respectively (17), 3.73 mPa⋅s of 20 wt% Ficoll (47)).

The addition of Mg^2+^ stabilizes the closed state by enhancing the folding rate, *k*_f_, and by decreasing the unfolding rate, *k*_u_: *k*_f_ increases from 7.5 ± 0.2 s^−1^ in neat buffer to 11.9 ± 0.3 s^−1^ and 13.3 ± 0.2 s^−1^ in the presence of 1 and 6 mM Mg^2+^, while *k*_u_ decreases from 3.13 ± 0.05 s^−1^ in neat buffer to 1.25 ± 0.02 s^−1^ and 1.65 ± 0.04 s^−1^ for 1 and 6 mM Mg^2+^, respectively.

On the contrary, TMAO exhibits a decrease in both *k*_f_ and *k*_u_. However, addition of high TMAO concentrations also leads to a significant enhancement in the solution viscosity, *η*. To obtain viscosity-corrected folding and unfolding rate constants, the folding and unfolding times obtained from the exponential fit to the dwell time histograms have to be corrected for the viscosity change, using $k_{{\rm{f}}( {\rm{u}} )}^{{\rm{corr}}} = \ k_{{\rm{f}}( {\rm{u}} )}^{{\rm{uncorr}}}\ ( {{\eta _{{\rm{sample}}}}/{\eta _{{\rm{buffer}}}}} )$ and published microviscosity data of 2 and 4 M TMAO (17,47,65); $k_{{\rm{f}}( {\rm{u}} )}^{{\rm{corr}}}$ and $k_{{\rm{f}}( {\rm{u}} )}^{{\rm{uncorr}}}$ are the corrected and uncorrected folding (unfolding) rate constants, respectively. The viscosity-corrected folding rates, $k_{\rm{f}}^{{\rm{corr}}},$ for 2 and 4 M TMAO are found to be 10.5 ± 0.4 s^−1^ and 23.2 ± 0.4 s^−1^, respectively. The corrected unfolding rate constant, $k_{{\rm{u}}}^{{\rm{corr}}}$, for 2 and 4 M TMAO are quite similar to the neat buffer data and amount to 2.39 ± 0.07 s^−1^ and 4.01 ± 0.16 s^−1^, respectively. Hence, TMAO strongly affects the folding rate constant, while the unfolding rate remains more or less the same with respect to that in neat buffer solution. This kinetic stabilizing mechanism found for TMAO is quite different from that of Mg^2+^, where both rate constants are affected.

Interestingly, apart from the fully open and closed state, different conformational states have been observed by the immobilized smFRET measurements. Almost 10% of the population in the presence of 2 M TMAO displays a three-state behavior as shown in Supplementary Figure S9. *E*-histograms corresponding to those states have *E*-values of 0.14 ± 0.01, 0.51 ± 0.01 and 0.78 ± 0.01. State 1 with *E* = 0.14 represents the fully open conformation, state 3 with *E* = 0.78 the fully closed state, in agreement with literature data (17,25). State 2 with *E* = 0.51 might represent a partially folded conformer where the stem is incompletely formed. All these states interconvert among themselves. However, according to the FRET time series shown in Supplementary Figure S9, we recognize that the most probable transitions are between state 3 and state 1 or state 3 and state 2. Only occasionally, transitions occur between state 2 and state 1. The dwell time distribution plots obtained from the HMM fits to the FRET time series (Supplementary Figure S7) for each of these states are shown in Supplementary Figure S10. The dwell times were corrected for the viscosity contribution and are used to obtain the corresponding rate constants (Table 3): *k*_1_ represents the transition rate mostly from the fully open state 1 to the fully closed state 3 conformer, and also to some extent to the partially folded conformer (state 2); *k*_2_ indicates mostly the transition rate from the partially folded conformer (state 2) to the fully closed state 3, and to a lesser extent to the fully open state 1; *k*_3_ provides the rate constant for the transition from the fully closed state 3 to the fully open or partially folded conformers (states 1, 2). Hence, *k*_1_ and *k*_2_ represent essentially folding and *k*_3_ unfolding rates. The values of *k*_1_ and *k*_2_ are much higher than *k*_3_, indicating that even though TMAO populates to some extent the partially folded state, it still favors complete folding and it strongly stabilizes the closed conformation of the DNA Hp.

**Table 3.** The dwell times obtained from the single exponential fits to the respective dwell time distribution plots in case of 2 M TMAO (Supplementary Figure S10) and 2 M urea (Supplementary Figure S12)

Parameters	2 M TMAO	2 M urea
${t_1}$ / ms	69.8 ± 7.4	87.1 ± 3.0
${t_2}$ / ms	101.0 ± 10.6	54.7 ± 3.0
${t_3}$ / ms	527.2 ± 28.0	54.3 ± 2.6
$t_1^{{\rm{corr}}}$/ ms	37.6 ± 1.8	
$t_2^{{\rm{corr}}}$/ ms	54.5 ± 5.7	
$t_3^{{\rm{corr}}}$/ ms	284.3 ± 15.1	
*k*_1_ / s^−1^	26.7 ± 1.3	11.5 ± 0.4
*k*_2_ / s^−1^	18.3 ± 2.0	18.3 ± 1.0
*k*_3_ / s^−1^	3.5 ± 0.2	18.4 ± 0.9

The rate constants in case of 2 M TMAO belong to the 10% population of the DNA Hp that displays a three-state behavior. In case of 2 M TMAO, the *t* and *k* values are corrected for the viscosity contribution.

Conversely and as expected, the addition of urea to the buffer solution destabilizes the closed state and favors the open state of the DNA Hp. This is already visible from the increasing amount of donor signal (green) in the fluorescence traces with increasing urea concentration (Figure 6D). Additional peaks in the *E*-histograms are observed for 2 M urea, apart from the two peaks due to the fully open (*E* = 0.14) and fully closed state (*E* = 0.85), indicating a more complex conformational landscape in the presence of urea. In case of urea, all the single-molecule traces display a three-state transition as shown in Supplementary Figure S11. The FRET efficiency distribution of state 1 is centered at *E* = 0.17 ± 0.01, of state 2 at *E* = 0.38 ± 0.01 and the state 3 has a bimodal FRET efficiency distribution with peak maxima centered at *E* = 0.51 ± 0.01 and *E* = 0.86 ± 0.01, respectively. This is because some molecules of the DNA Hp in the presence of 2 M urea do not exhibit the state with *E* ≈ 0.85 but rather show a three-state dynamics corresponding to the states with *E* ≈ 0.5, 0.3 and 0.2. Hence, for the state 3 a bimodal FRET distribution has been assigned. The kinetic constants between these states are obtained from the dwell time distribution plots (Supplementary Figure S12) and are displayed in Table 3. The rate constant *k*_1_ represents the dynamics for the transition from the fully open state 1 to the partially unfolded state 2 and folded state 3; *k*_2_ indicates the dynamics from the partially unfolded state to either the fully open or the fully closed state and *k*_3_ dictates the rate at which the fully closed state transforms to the partially folded and fully open states, i.e. represents the unfolding dynamics in the presence of 2 M urea. The *k*_3_ value of 18.4 s^−1^ is much higher than the unfolding rate of *k*_u_ = 3.13 s^−1^ found in neat buffer. Hence, 2 M urea populates partially unfolded and fully open states by increasing the unfolding rate constant, while interfering little with the folding rate *k*_1_ in comparison to the scenario in neat buffer solution. In case of 4 M urea, ∼92% of the population has shifted to the fully open state (*E* = 0.17) already, only a tiny fraction of the molecules remains in a state with *E* = 0.51, which represents a partially folded conformer. The folding rate constant *k*_f_ determined in case of 4 M urea from the dwell time of the state with *E* = 0.17 amounts to 1.94 ± 0.03 s^−1^, which is much lower than the value of 7.5 s^−1^ observed in neat buffer (Supplementary Figure S13, Table 2). On the other hand, the value of *k*_u_ for 4 M urea determined from the dwell time of the state with *E* = 0.51 is found to be 14.5 ± 0.5 s^−1^, which is again faster than the value of *k*_u_ = 3.13 s^−1^ found in neat buffer. Hence, 4 M urea stabilizes the fully open conformation of the DNA Hp by increasing the *k*_u_ and decreasing the *k*_f_ value concomitantly.

The introduction of the crowding agent 20 wt% Ficoll leads also to the formation of additional peaks in the *E*-histograms for the DNA Hp (Figure 6E). Peak maxima are recorded at *E* = 0.13 ± 0.01, 0.31 ± 0.01, 0.60 ± 0.01 and 0.85 ± 0.01, pointing to an even more rugged conformational landscape of the DNA Hp. In case of 20 wt% Ficoll, the single-molecule fluorescence traces display mostly two-state dynamics as shown in Supplementary Figure S14. We may consider the two-state dynamics in terms of folded and unfolded states with a bimodal FRET distribution each. The FRET distribution in case of the folded state is centered at *E* = 0.60 ± 0.01 and 0.85 ± 0.01, and that of the unfolded state at *E* = 0.13 ± 0.01, 0.31 ± 0.01, respectively. The dwell times were corrected for the increased viscosity in 20 wt% Ficoll solution and used to determine the folding ($k_{\rm{f}}^{{\rm{corr}}}$) and unfolding ($k_{\rm{u}}^{{\rm{corr}}}$) rate constants. The $k_{\rm{f}}^{{\rm{corr}}}$ value is found to be 48.9 ± 0.1 s^−1^, which is drastically faster than the value of 7.5 ± 0.2 s^−1^ observed for neat buffer (Table 3). The $k_{\rm{u}}^{{\rm{corr}}}$ value of 4.13 ± 0.08 s^−1^ observed in the presence of 20 wt% Ficoll does not differ much from the neat buffer value of 3.13 ± 0.05 s^−1^, however (Table 2). Hence, Ficoll stabilizes the folded state ensemble kinetically by markedly enhancing the folding rate.

## DISCUSSION

The volumetric profile for a conformational transition depends strongly on the packing arrangement of the structural units in their respective conformational state and is very sensitive to hydration changes. The partial molar volume, *V*^o^, of a solute in solution can be decomposed into a sum of following contributions (11,16,66):

(1) \begin{equation*}{V^{\rm{o}}} = {V_{\rm{M}}} + {V_{\rm{T}}} + {V_{\rm{I}}},\end{equation*}

where *V*_M_ is the molecular volume, i.e. the geometric volume impenetrable to water molecules, *V*_T_ is the thermal volume indicating the void space surrounding the solvent accessible surface area (SASA) resulting from mutual vibrations and librations of the solute and solvent molecules and *V*_I_ represents the interaction volume indicating interfacial hydration changes. For an unfolding transition of the DNA Hp, the volume change, Δ*V*^o^, is given by the sum of the molecular, thermal and interaction volume changes (12,16,18,19,67,68):

(2) \begin{equation*}\Delta {V^{\rm{o}}} = \Delta {V_{\rm{M}}} + \Delta {V_{\rm{T}}} + \Delta {V_{\rm{I}}}.\end{equation*}

Generally, Δ*V*_M_ < 0 arises only from a release of void volume (originating in an imperfect packing) upon unfolding. For a perfectly matched DNA duplex structure, the change in molecular volume Δ*V*_M_ is typically assumed negligible due to the absence of any internal voids (14). However, Son et al. have found a negative volume change for Δ*V*_M_ upon unfolding of a DNA duplex (12). Nevertheless, in the case of DNA duplex unfolding, all the volume contributions cancel each other largely, leading to a very small positive volume change, which is similar to the volume of one H_2_O molecule, only (12). Δ*V*_T_ is proportional to the SASA and is always positive for unfolding transitions. The change of interaction volume, Δ*V*_I_, represents the hydration volume change resulting from the difference between water molecules in the denser hydration layer around the biomolecule and the bulk phase. The partial molar volume of water in the hydration shell is ∼10% smaller than the volume of water in the bulk water phase (12,16,69), which is due to the contraction of water around polar and charged groups of the biomolecule (electrostriction effect). For an unfolding transition, the change in interaction volume can be represented by Δ*V*_I_ = Δ*n*_h_(*V*_h_–*V*_o_), where Δ*n*_h_ is the number of water molecules taken up or released during the unfolding transition, *V*_h_ and *V*_o_ are the partial molar volumes of water in the hydration shell and the bulk phase, respectively (12,16). Consequently, an increase in hydration upon unfolding leads always to an overall negative volume change.

**Figure 7.**
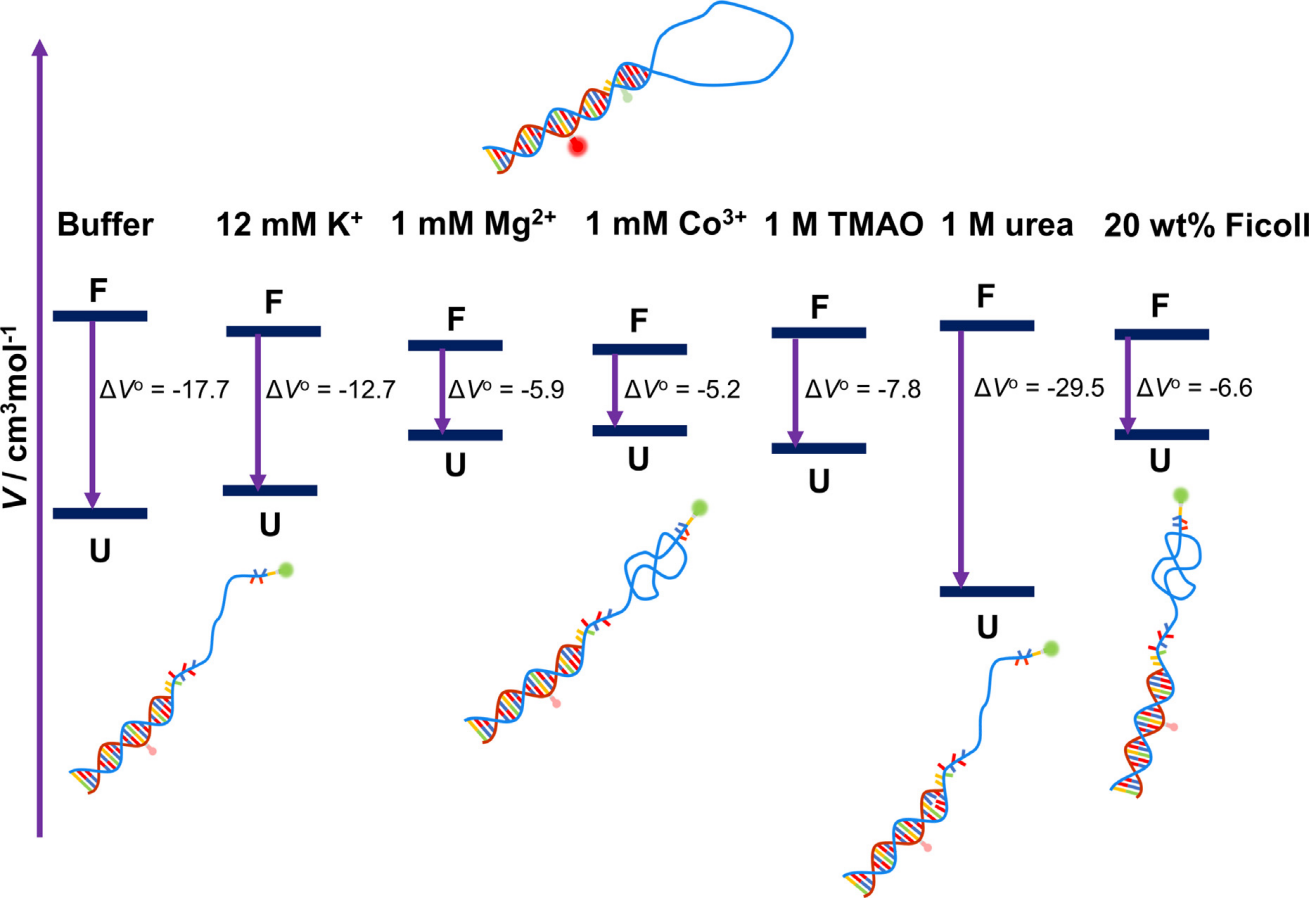
Schematic representation of the volumetric profile of the DNA Hp at 25°C for the unfolding transition in neat buffer and in the presence of salts (K^+^, Mg^2+^ and Co^3+^), osmolytes (TMAO, urea) and the crowding agent Ficoll, based on the results listed in Table 1. U and F represent the unfolded and folded states, respectively, and Δ*V*^o^ = *V*^o^_u_ - *V*^o^_f_ the partial molar volume change upon unfolding. In the presence of salts, TMAO and Ficoll, the unfolded states are more collapsed, leading to a lower SASA, to a smaller hydration contribution, and hence an overall larger volume compared to *V*^o^_u_ in neat buffer. Conversely, in the presence of 1 M urea, the unfolded state becomes more extended. The larger SASA leads to a strong hydration contribution and hence an overall smaller volume in comparison to the unfolded state in neat buffer. Please note that the volume changes are very small (for comparison, one H_2_O molecule has a molar volume of 18 cm^3^mol^−1^). The diagram is not to scale. Relative positions of Δ*V*^o^ should be viewed as qualitative, only.

In neat buffer, pressurization shifts the conformational equilibrium of the DNA Hp towards the unfolded state (Figures 2A and 4B). The volume change upon unfolding amounts to −17.7 ± 1.7 cm^3^mol^−1^, indicating a lower molar volume of the unfolded state than the folded state for the polydA loop DNA Hp (Figure 7). Generally, such negative volume change is attributed to the hydration volume change upon unfolding as the unfolded state has a larger SASA than the folded state (12,16,19). For a smaller loop DNA Hp, the Δ*V*^o^ has been found to be in the range of −2.35 to +6.74 cm^3^mol^−1^, depending on the salt concentration and on temperature (67). A simulation study on a small loop RNA hairpin estimated the Δ*V*^o^ upon pressure-induced unfolding to be about −4 cm^3^mol^−1^ (70). This suggests that packing defects of the bases in the folded structure of our DNA Hp (i.e. void volume which is filled with water upon unfolding) contributes to Δ*V*^o^ as well.

Upon addition of salt, in particular the divalent Mg^2+^ and trivalent Co^3+^, Δ*V*^o^ decreases and strongly counteracts the destabilization effect of pressure on the folded state (Figure 7). The addition of salt reduces the repulsive interaction between the backbone’s phosphate groups of the DNA Hp by charge screening, resulting in more compact packing and smaller void volume in the folded state and, probably more significantly, a more compact state with smaller SASA for the unfolded state in comparison to the neat buffer scenario (Figure 7). This effect increases with increasing charge density (Co^3+^ > Mg^2+^ > K^+^). Hence, the unfolded state in the presence of salt will have a lower SASA and lower hydration contribution, resulting in an overall larger volume compared to the unfolded state in neat buffer (Figure 7).

The addition of TMAO and Ficoll also populates the high-FRET state and counteracts the pressure effect by rendering the unfolding process volumetrically less favorable (Table 1). The interaction of TMAO with the phosphate groups and also with the nucleobases of nucleic acids has been found to be strongly unfavorable (9,23,71,72). This results in an exclusion of TMAO from the surface of the DNA Hp and a compaction of the DNA Hp structure with minimal SASA. The preferential exclusion effect is particularly strong in case of the unfolded state, which leads to a compact unfolded state (ensemble) with overall larger volume owing to a smaller hydration contribution (Figure 7). In addition, the folded state in the presence of TMAO might have a somewhat smaller volume with lower void volume due to the strong excluded volume effect as well. On the other hand, the crowding agent Ficoll (radius ∼5.5 nm), due to its larger size, reduces the available space in the solvent for the biomolecules to occupy (61,62). As a result of this strong steric excluded volume effect, the most compact conformation of the DNA Hp is favored. Hence, the effect on the unfolded state structure is significantly larger than on the folded state. Overall, the presence of Ficoll leads to a smaller volume change upon unfolding as presented schematically in Figure 7.

On the contrary, addition of urea renders the pressure-induced unfolding process of the DNA Hp volumetrically more favorable, i.e. it synergistically destabilizes the folded state of the DNA Hp at high pressures (Figures 4C and 5C). The Δ*V*° changes from −17.7 cm^3^mol^−1^ in neat buffer to −29.5 cm^3^mol^−1^ in 1 M urea. Urea interacts favorably with the nucleobases and therefore favors extended conformations with large SASA (17,23,73,74). As a result, the unfolded state is strongly hydrated, leading to a smaller volume in comparison to the unfolded state in neat buffer (Figure 7). Urea also weakens base stacking interactions, which may result in breaking of a few base pairs between the two complementary strands, which leads to a stronger hydration and a more negative hydration volume change.

**Figure 8.**
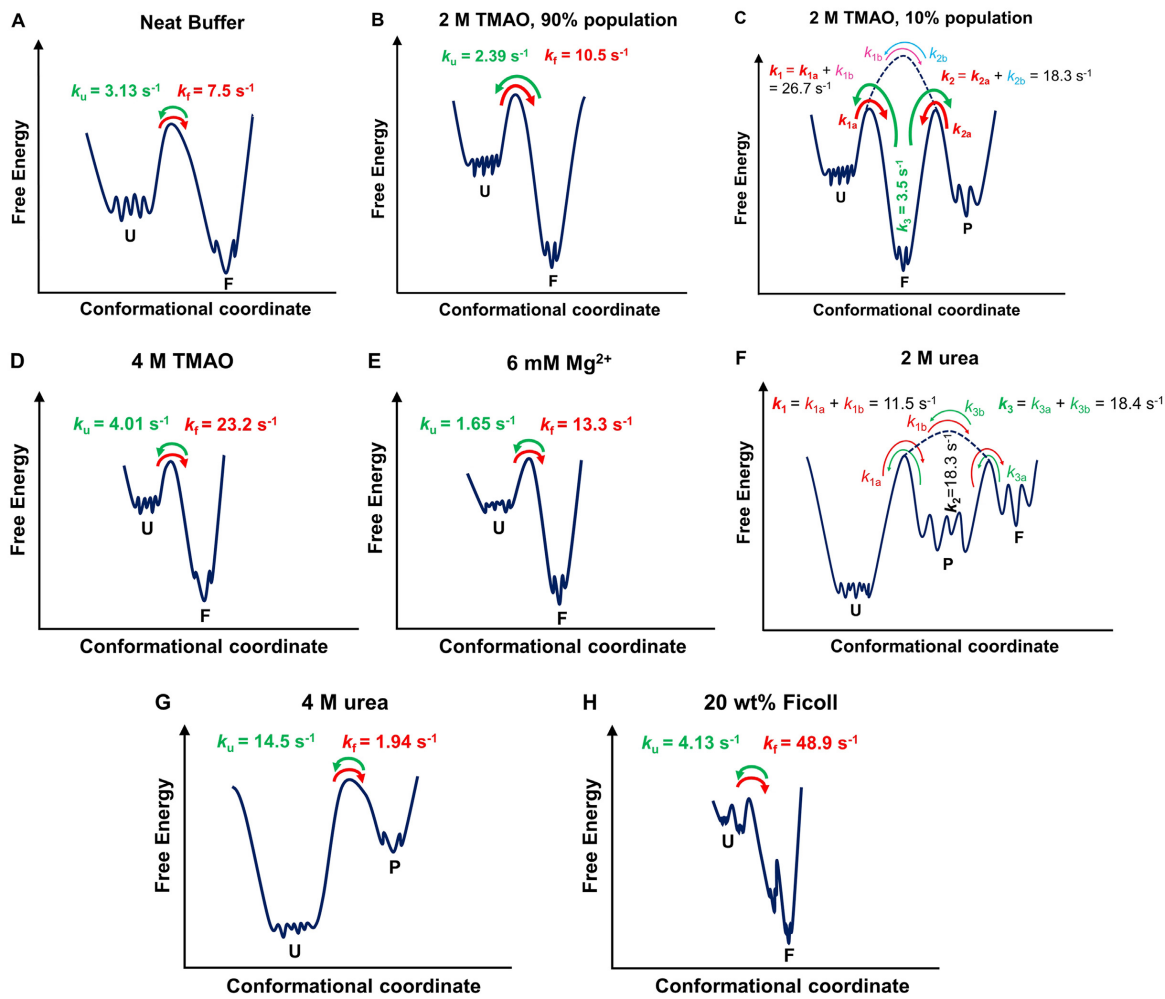
Schematic of the free energy landscape of the DNA Hp in (**A**) neat buffer, (**B** and **C**) 2 M TMAO, (**D**) 4 M TMAO, (**E**) 6 mM Mg^2+^, (**F**) 2 M urea, (**G**) 4 M urea and (**H**) 20 wt% Ficoll. U, F and P denote unfolded, folded and partially folded conformations. In the case of 2 M TMAO, the two schematics describe the two-state dynamics observed for 90% of the population of states observed, and three-state dynamics displayed by the remaining 10% of the population. In (a) neat buffer, (b) 90% population in 2 M TMAO, (d) 4 M TMAO, (e) 6 mM Mg^2+^ and (g) 4 M urea, a two-state dynamics is observed where the states are separated by a distinct free energy barrier. Green and red curved arrows display the unfolding and folding dynamics, respectively, *k*_u_ and *k*_f_ denote the corresponding unfolding and folding rate constants. A more complex conformational landscape is observed in the case of (c)10% population of 2 M TMAO, (f) 2 M urea and (h) 20 wt% Ficoll. In the case of 2 M TMAO, 10% of the population exhibits a three-state behavior, indicating the presence of a relatively stable partially folded state (P) along with U and F. As can be seen in Figure S9 (SI), most of the transitions in 2 M TMAO occur between U and F or F and P (indicated by thick red and green curved arrows), only occasionally transitions occur between U and P (indicated by thin purple and blue curved arrows). Hence, in 2 M TMAO *k*_1_ signifies the dynamics from U to mostly F (red curved arrow) and occasionally to P (thin purple curved arrow), *k*_2_ represents the transition rate from P to mostly F (red curves arrow) and occasionally to U (thin blue curved arrow) and *k*_3_ indicates the transition from F to either P or U. Hence, for the 10% population in 2 M TMAO, *k*_1_ and *k*_2_ essentially represent folding and *k*_3_ unfolding rates. A three-state behavior is also observed in case of 2 M urea (e). Here, *k*_1_ represents the transition rate from U to either F or P, *k*_2_ from P to either U or F and *k*_3_ the transition from F to U or P. Thus, *k*_3_ indicates the rate of unfolding, which is much larger compared to neat buffer. For 20 wt% Ficoll (g), two kinds of unfolded and folded states have been identified, which may still be described by an overall two-state dynamics where each of the unfolded states converts to a folded state and vice versa.

The kinetic profile of the conformational dynamics of the DNA Hp in neat buffer and in the presence of cosolvents and crowding media obtained from the smFRET measurements under immobilized conditions provide deeper insights into cosolute effects on the folding energy landscapes (Figure 8). In neat buffer, a two-state conformational dynamics is obtained (Figure 6A) with folding (*k*_f_) and unfolding (*k*_u_) rates of 7.5 and 3.13 s^−1^, respectively which are similar to the ambient-pressure literature data (25,75). Two-state dynamics is also observed in the case of 2 M TMAO (90% population), 4 M TMAO, Mg^2+^ and 4 M urea (Figures 6 and 8). Increasing concentrations of TMAO and Mg^2+^ strongly stabilize the folded state, however, the mechanism of this stabilization seems to be different. In case of TMAO, *k*_f_ is strongly enhanced, while *k*_u_ does not differ much from the value observed in neat buffer (Table 2). Addition of Mg^2+^ affects both, *k*_f_ and *k*_u_. Mg^2+^ leads to an effective charge screening of the phosphate backbone, thereby enhancing *k*_f_ and reducing *k*_u_. Mg^2+^ is also strongly hydrated and this might decrease the number of water molecules surrounding the DNA Hp, favoring a structure with lowest possible SASA, i.e. the folded state. On the other hand, TMAO stabilizes the folded structure due to a pronounced excluded volume effect, owing to its unfavorable interaction with both the phosphate and nucleobase backbone (23,68,71). Conversely, urea leads to a significant enhancement of *k*_u_ and a drop of *k*_f_ due to its favorable interaction with the nucleobases, and therefore stabilizes structures with larger nucleobase SASA, i.e. the unfolded state (73,74,76).

Interestingly, we found that cosolvents and crowding can populate different conformational substates to some extent that are not encountered in the case of neat buffer solution. Indications for a more rugged free energy landscapes for the DNA Hp with stable or metastable intermediates can be found in the literature as well (17,36,37,39,40). We showed that in the presence of 2 M TMAO, almost 10% of the population visits a third substate (P) with a different *E-*value (*E* = 0.51) next to the fully open (U) and closed state (F). Such partially folded state is most likely a conformer where the stem is incompletely formed, but which displays an excluded-volume-induced low-SASA structure, already. Figure 8 depicts a schematic of the free energy landscape of the DNA Hp and how it is modulated by the cosolutes. In case of 2 M TMAO, the most probable transitions occur between U (*E* ≈ 0.2) and F (*E* ≈ 0.8) or P (*E* ≈ 0.5) and F. Only occasionally, a transition is observed between U and P (Supplementary Figure S9). Hence, for the 10% population in 2 M TMAO, *k*_1_ and *k*_2_ represent essentially the folding dynamics from the unfolded (U) and partially folded ensembles (P), which are found to be 26.7 and 18.3 s^−1^, respectively, and which are much larger than the value of *k*_f_ = 7.5 s^−1^ found in neat buffer, while *k*_3_ specifies the unfolding dynamics and amounts to 3.5 s^−1^, a value very similar to the value of *k*_u_ = 3.13 s^−1^ in neat buffer. Thus, both the unfolded and partially folded states tend to give way to the folded state, which is the most stable conformation in the presence of TMAO as it has the lowest SASA (Figure 8). Two different unfolded ensembles with distinct refolding times have also been observed using temperature- and ion-jump perturbation techniques (37). According to literature reports for this class of DNA Hp, the unfolded basin is very broad and flat with many local minima while the folded state occupies minima of a narrow funnel (37). Configuration diffusion on this rather flat energy surface characterizes the dynamics within the unfolded states, which fold with distinct folding times from different regions of the conformational landscape. In the presence of TMAO, compact conformations are favored and might be trapped in an intermediate state denoted P, which could be a partially folded state where the stem of the hairpin is incompletely formed. This might be the reason why 10% of the population displays a three-state dynamics in the presence of 2 M TMAO.

A rugged energy landscape with a multitude of partially folded conformational states is also observed in the presence of 2 M urea (Figure 6D). Urea interacts strongly with nucleobases, thereby weakening base stacking interactions and H-bonding, leading to the formation of partially folded conformers. The rate constant *k*_3_ in the presence of 2 M urea indicates unfolding of the closed state to partially folded (P) and unfolded (U) conformations, and amounts to 18.4 s^−1^, which is much higher compared to *k*_u_ = 3.13 s^−1^ in neat buffer. The formation of partially folded states is also observed in case of 4 M urea. The high-FRET state (with *E* value of 0.51) indicates a partially folded conformation, only (Figure 6D).

Also in the presence of Ficoll, formation of additional conformational substates is observed (Figure 6E). Although we found a two-state dynamics in the presence of Ficoll (Supplementary Figure S14), both the folded and unfolded states exhibit a bimodal FRET distribution (Figure 6E), indicating two different kinds of unfolded and folded conformers (Figure 8). The unfolded state in 20 wt% Ficoll with *E* = 0.13 and *E* = 0.31 represents the fully open state and, most likely, a conformation where only one or two base pairs in the stem are formed (Figure 8). The folded conformer with *E* = 0.60 suggests a partially folded structure where a stable but yet incomplete stem is formed (Figure 8). Due to a strong excluded volume effect, eventually in concert with soft ‘quinary’ enthalpic interactions between the crowder and DNA HP, such additional compact structures may have formed. The underlying kinetic stabilization mechanism is very similar to the TMAO case. The strong excluded volume effect drastically enhances the *k*_f_ value (*k*_f_ = 48.9 s^−1^), while *k*_u_ = 4.13 s^−1^ does not change much in comparison to neat buffer (Table 2).

## CONCLUSIONS

To conclude, we have performed smFRET measurements in the presence of different biologically relevant salts, cosolvents and crowding media, mimicking intracellular conditions, to deduce their impact on the volumetric and kinetic profile of the conformational dynamics of a DNA Hp. The results provide novel mechanistic insights into the conformational landscape of this class of biomolecule and how it is affected by stressors, including pressure. Using pressure modulation, the volumetric properties of the various conformational states of the DNA Hp could be exploited. We found pressurization to shift the conformational equilibrium towards the unfolded state, which occupies a smaller partial molar volume. Addition of salt, TMAO and Ficoll counteracts the pressure-induced destabilization of the folded conformation by rendering the unfolding process volumetrically less favorable. The salt effect is particularly strong at high charge density of the cation (Co^3+^ > Mg^2+^ > K^+^). Conversely, urea synergistically destabilizes the folded state with pressure, as the unfolding process becomes volumetrically more favorable. The results can be rationalized by invoking changes in hydration and intrinsic void volume of the conformers involved. The smFRET measurements under immobilized conditions provided the underlying rate constants for the various conformational transitions, providing also detailed insights into the mechanism dictating the conformational preference of the biomolecule at the different solution conditions. Furthermore, these data provide compelling evidence of a more rugged energy and conformational landscape in the presence of osmolytes and crowders. The dissection into volumetric and kinetic parameters at different solution conditions advances our understanding of the impact of the complex cellular milieu on the conformational dynamics of biomolecules such as the DNA Hp studied here. We believe that the stabilizing or destabilizing effect of the cosolutes in terms of volumetric and kinetic properties will be similar to other hairpin systems. Our data also demonstrate the important role of compatible osmolytes and cellular crowding in rescuing biological function under harsh environmental conditions, such as at HHP even of the 1000 bar-level. Equilibrium and kinetic parameters at high-pressure conditions are controlled by the cosolute’s impact on the volumetric properties of the dissolved biomolecule.

## Supplementary Material

Supplementary Data
