# Supplementary material for: Exploring the effects of cosolutes and crowding on the volumetric and kinetic profile of the conformational dynamics of a poly dA loop DNA hairpin: a single-molecule FRET study
Source: Nucleic Acids Res. 2018 Nov 12;47(2):981–96. doi: 10.1093/nar/gky1122 (PMC6344865; doi:10.1093/nar/gky1122)
Supplement: Supplementary Data [file gky1122_supplemental_files.pdf]

Exploring the effect of cosolvents and crowding on the volumetric and kinetic profile of the conformational dynamics of a poly dA loop DNA Hairpin: a single-molecule FRET study

Satyajit Patra[1], Vitor Schuabb[1], Irena Kiesel[1], Jim Marcel Knop[1], Rosario Oliva[1], and Roland Winter[1*]

[1] Physical Chemistry I - Biophysical Chemistry, Faculty of Chemistry and Chemical Biology, TU Dortmund University, Otto-Hahn Street 4a, 44227 Dortmund, Germany, [2] Department of Chemical Sciences, University of Naples Federico II, Via Cinita, 80126 Naples, Italy

*E-mail: roland.winter@tu-dortmund.de

Contents:

1. Results of the smFRET measurement on the DNA Hp at different pressures obtained for different concentrations of Co^{3+} (**Figure S1**).

2. Plot of lnK_{eq} vs. p in neat buffer and in the presence of K^+ and Mg^{2+} (**Figure S2**).

3. Ensemble FRET measurement in buffer in the absence and presence of different concentrations of K^+ and Mg^{2+} (**Figures S3 and S4**).

4. Plot of lnK_{eq} vs. p in neat buffer and in the presence of 1 M TMAO, 1 M urea and 20 wt% Ficoll (**Figure S5**).

5. Normalized fluorescence spectra of the Atto 550 and Atto 647N labelled DNA Hp at different pressures in neat buffer and different concentrations of Ficoll (**Figure S6**).

6. Pictorial description to visualize the Hidden Markov Model (HMM) analysis (**Figure S7**).

7. Cumulative dwell time (t) distribution plot in neat buffer and in the presence of different concentrations of TMAO and Mg^{2+} (**Figure S8**).

8. Representative single-molecule fluorescence traces of the Atto 550 and Atto 647N labelled DNA Hp in 2 M TMAO exhibiting a three-state behaviour (**Figure S9**).

9. Cumulative dwell time (t) distribution plot of the three states obtained for the 10% population of the DNA Hp in 2 M TMAO (**Figure S10**).

7. Example of the donor acceptor fluorescence traces of the dually labelled DNA Hp and their corresponding FRET time series obtained in case of 2 M urea (**Figure S11**).

8. Cumulative dwell time distribution plot for the three states found from the HMM fit to the FRET time series obtained for 2 M urea (**Figure S12**).

9. Cumulative dwell time distribution plot for the folding and unfolding of the DNA Hp at 4 M urea (**Figure S13**).

10. Representative examples of single-molecule fluorescence traces of the DNA Hp in the presence of 20 wt% Ficoll exhibiting a two-state behaviour (**Figure S14**).

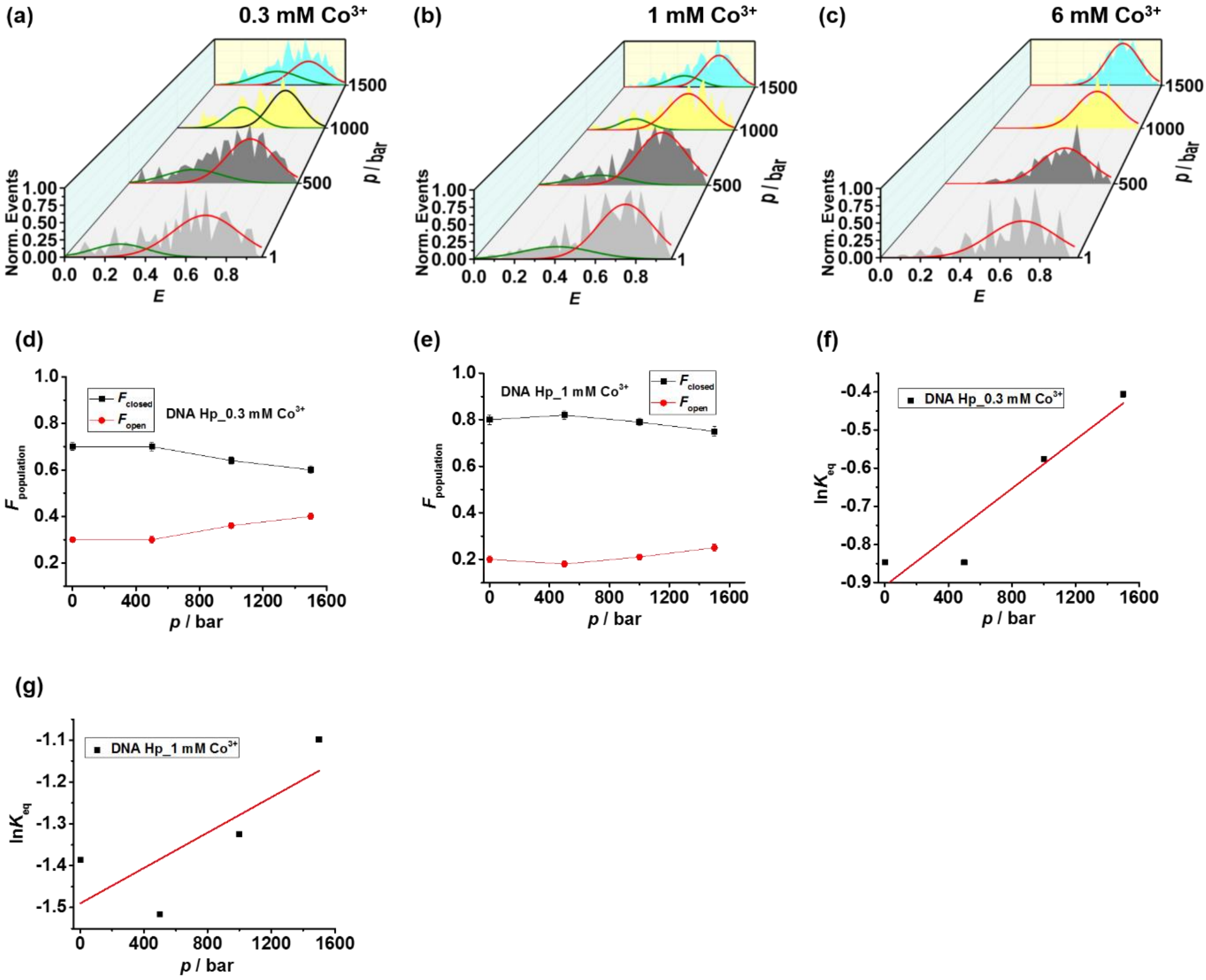

Figure S1. FRET efficiency (E) histograms of the DNA Hp in buffer (20 mM Tris HCl, 15 mM NaCl, pH = 7.5) with (a) 0.3 mM Co^{3+}, (b) 1 mM Co^{3+}, (c) 6 mM Co^{3+}. Green and red curves represent Gaussian fits to the data representing two different conformational states. Population at each conformational state determined from the peak areas of the Gaussian fits. The thus determined fractions of the population of the open (F_{open}) and closed (F_{closed}) state are plotted as a function of pressure in (d) 0.3 mM Co^{3+}, (e) 1 mM Co^{3+}. Plot of $\ln K_{eq}$ vs. p in (f) 0.3 mM Co^{3+} and (g) 1 mM Co^{3+} respectively. K_{eq} is the equilibrium constant for unfolding, defined as $K_{eq} = F_{open}/F_{closed}$. From the slope of the $\ln K_{eq}$ vs. p plot, the transition volume for unfolding (ΔV^{o}) is roughly estimated.

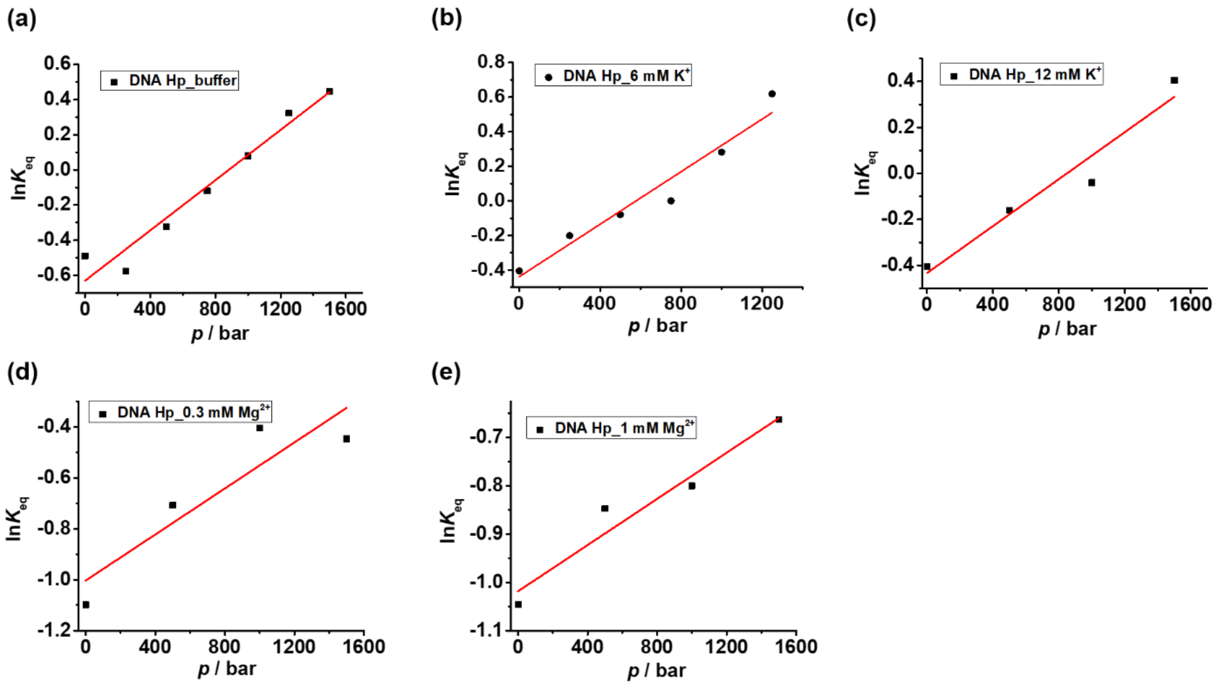

Figure S2. Plot of lnK_{eq} vs. p in (a) buffer, (b) 6 mM K^+, (c) 12 mM K^+, (d) 0.3 mM Mg^{2+} and (e) 1 mM Mg^{2+} respectively. K_{eq} is the equilibrium constant for unfolding, defined as $K_{eq} = F_{open}/F_{closed}$. From the slope of the lnK_{eq} vs. p plot, the transition volume for unfolding (ΔV^o) is determined.

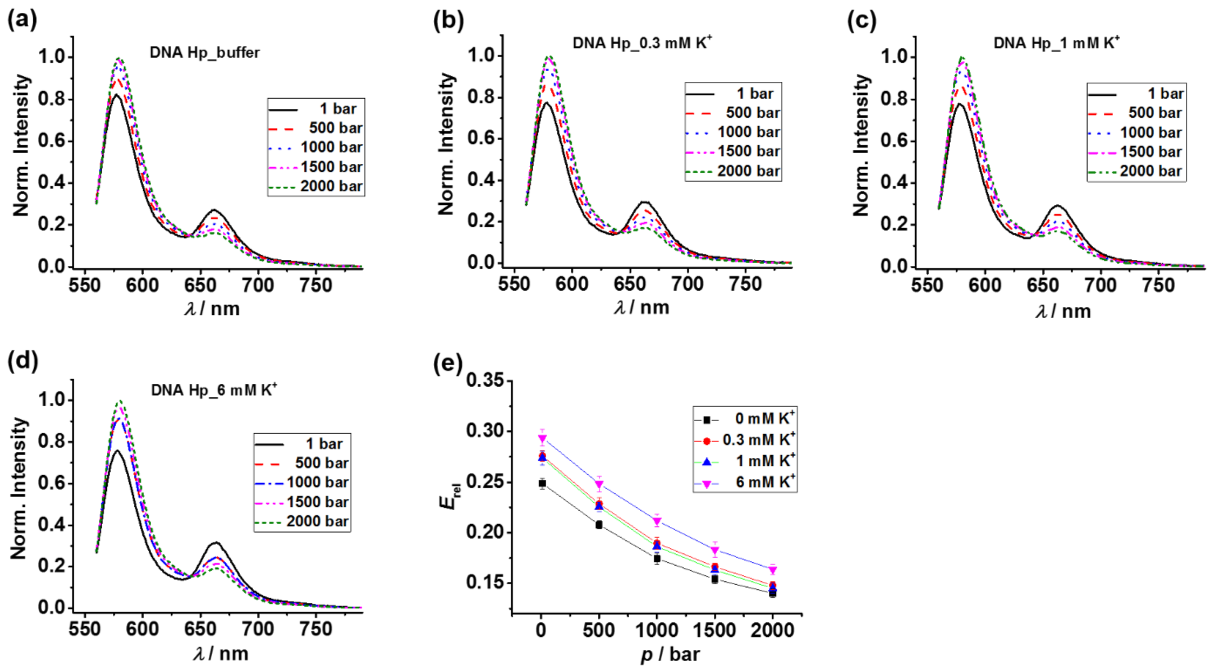

Figure S3. Normalized fluorescence spectra of the Atto 550 and Atto 647N labelled DNA Hp at different pressures in (a) neat buffer, (b) 0.3 mM K^+, (c) 1 mM K^+ and (d) 6 mM K^+ respectively. The excitation wavelength was 550 nm. The two peaks obtained in the emission spectra represent the donor and acceptor fluorescence, respectively. The emission peak at 580 nm represents fluorescence of the donor (Atto 550) and the peak at 664 nm indicates fluorescence of the acceptor (Atto 647N) due to FRET. (e) Plot of the relative FRET efficiency (E_{rel}), defined as $E_{rel} = I_A/(I_D+I_A)$, as a function of pressure. Here, I_D and I_A represent the donor and acceptor fluorescence intensities at their peak maxima, respectively. Increasing pressure leads to a decrease in the E_{rel}. K^+ up to a concentration of 6 mM has a negligible effect in stabilizing the closed state of the DNA Hp against pressure.

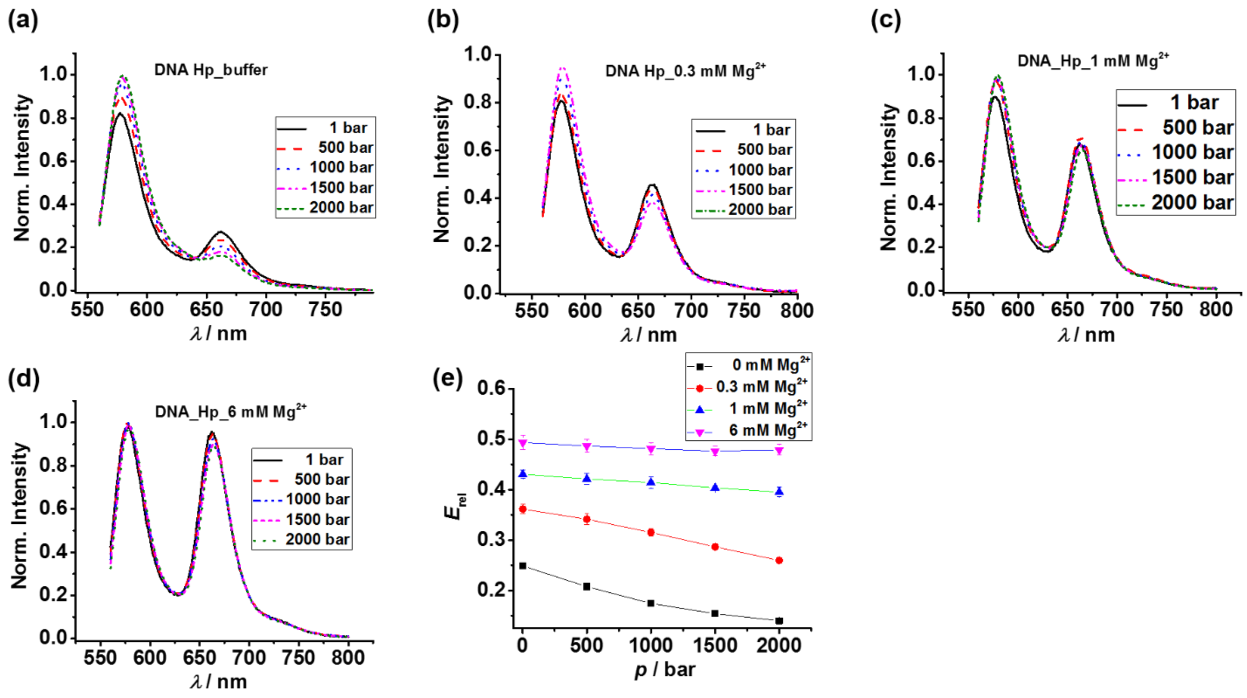

Figure S4. Normalized fluorescence spectra of the Atto 550 and Atto 647N labelled DNA Hp at different pressures in (a) neat buffer, (b) 0.3 mM Mg^{2+}, (c) 1 mM Mg^{2+} and (d) 6 mM Mg^{2+} respectively. The excitation wavelength was 550 nm. The two peaks obtained in the emission spectra represent the donor and acceptor fluorescence. The emission peak at 580 nm represents the fluorescence of the donor (Atto 550) and the peak at 664 nm indicates the fluorescence of the acceptor (Atto 647N) due to FRET. (e) A plot of the relative FRET efficiency (E_{rel}), defined as $E_{\mathrm{rel}} = I_{\mathrm{A}}/(I_{\mathrm{D}}+I_{\mathrm{A}})$, as a function of pressure. Here, I_{D} and I_{A} represent the donor and acceptor fluorescence, respectively. Increasing pressure leads to a decrease in E_{rel} in neat buffer. Conversely, increasing concentration of Mg^{2+} leads to an enhancement in the E_{rel} value and also counteracts the pressure-induced destabilization of the closed state.

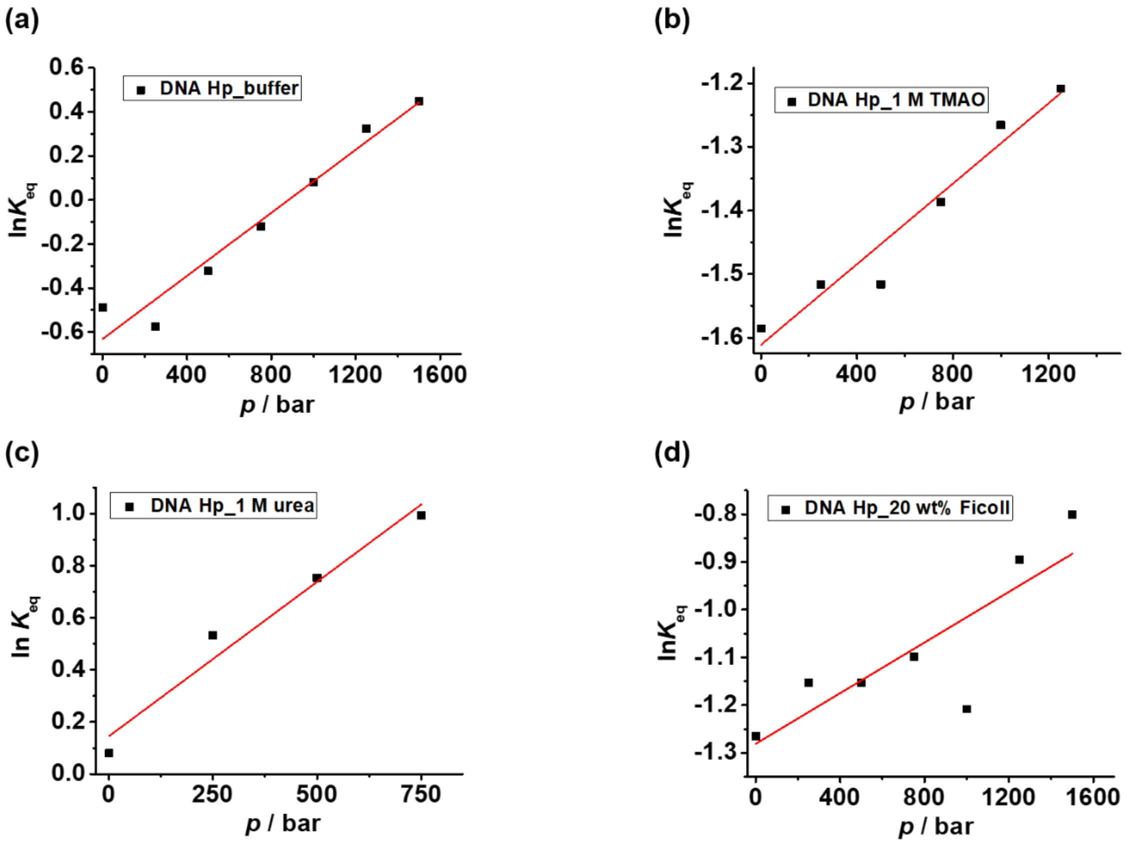

Figure S5. Plot of lnK_{eq} vs. p in (a) buffer, (b) 1 M TMAO, (c) 1 M urea and (d) 20 wt% Ficoll respectively. K_{eq} is the equilibrium constant for unfolding, defined as $K_{eq} = F_{open}/F_{closed}$. From the slope of the lnK_{eq} vs. p plot, the transition volume for unfolding (ΔV^{o}) is determined.

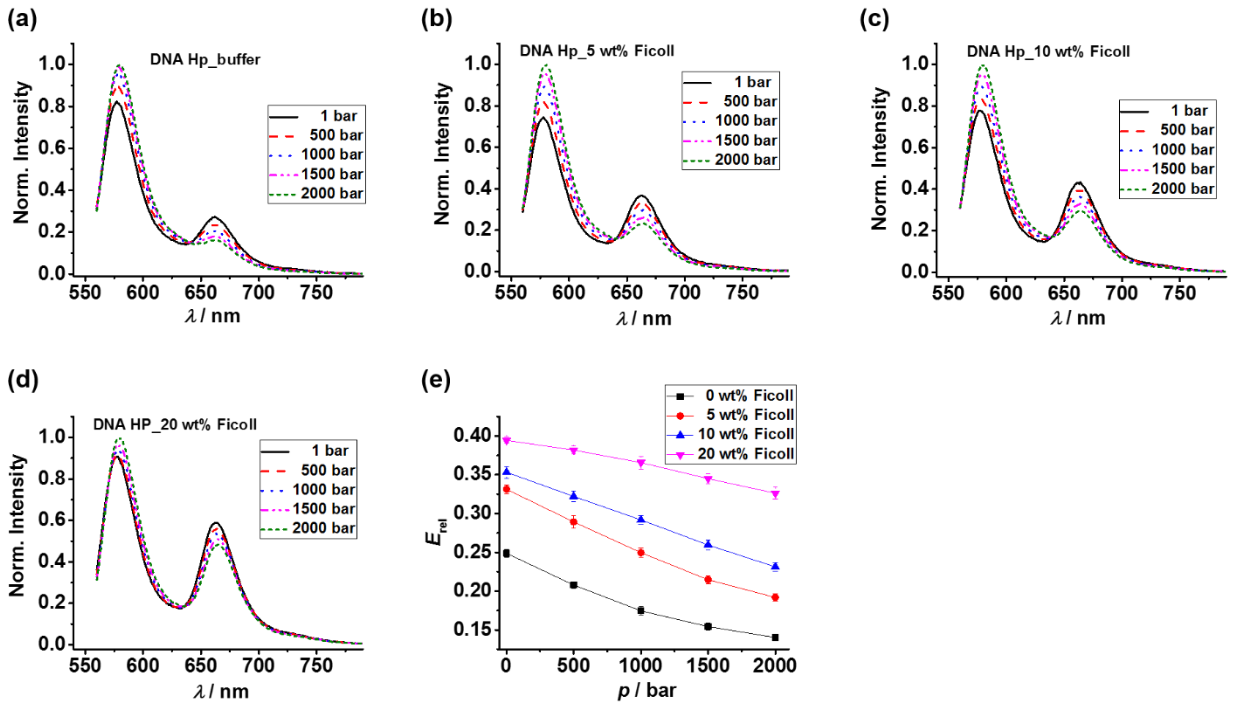

Figure S6. Normalized fluorescence spectra of the Atto 550 and Atto 647N labelled DNA Hp at different pressures in (a) neat buffer, (b) 5 wt% Ficoll, (c) 10 wt% Ficoll, and (d) 20 wt% Ficoll. The excitation wavelength was 550 nm. The two peaks obtained in the emission spectra represent the donor and acceptor fluorescence. The emission peak at 580 nm represents the fluorescence of the donor (Atto 550) and the peak at 664 nm indicates the fluorescence of the acceptor (Atto 647N) due to FRET. (e) Plot of the relative FRET efficiency (E_{rel}), defined as $E_{\mathrm{rel}} = I_{\mathrm{A}}/(I_{\mathrm{D}}+I_{\mathrm{A}})$, as a function of pressure. Here, I_{D} and I_{A} represent the donor and acceptor fluorescence, respectively. Increasing pressure leads to a decrease in the E_{rel} value in neat buffer. However, increasing concentrations of Ficoll lead to an enhancement in E_{rel} and also counteract the pressure-induced destabilization of the closed state of the DNA Hp.

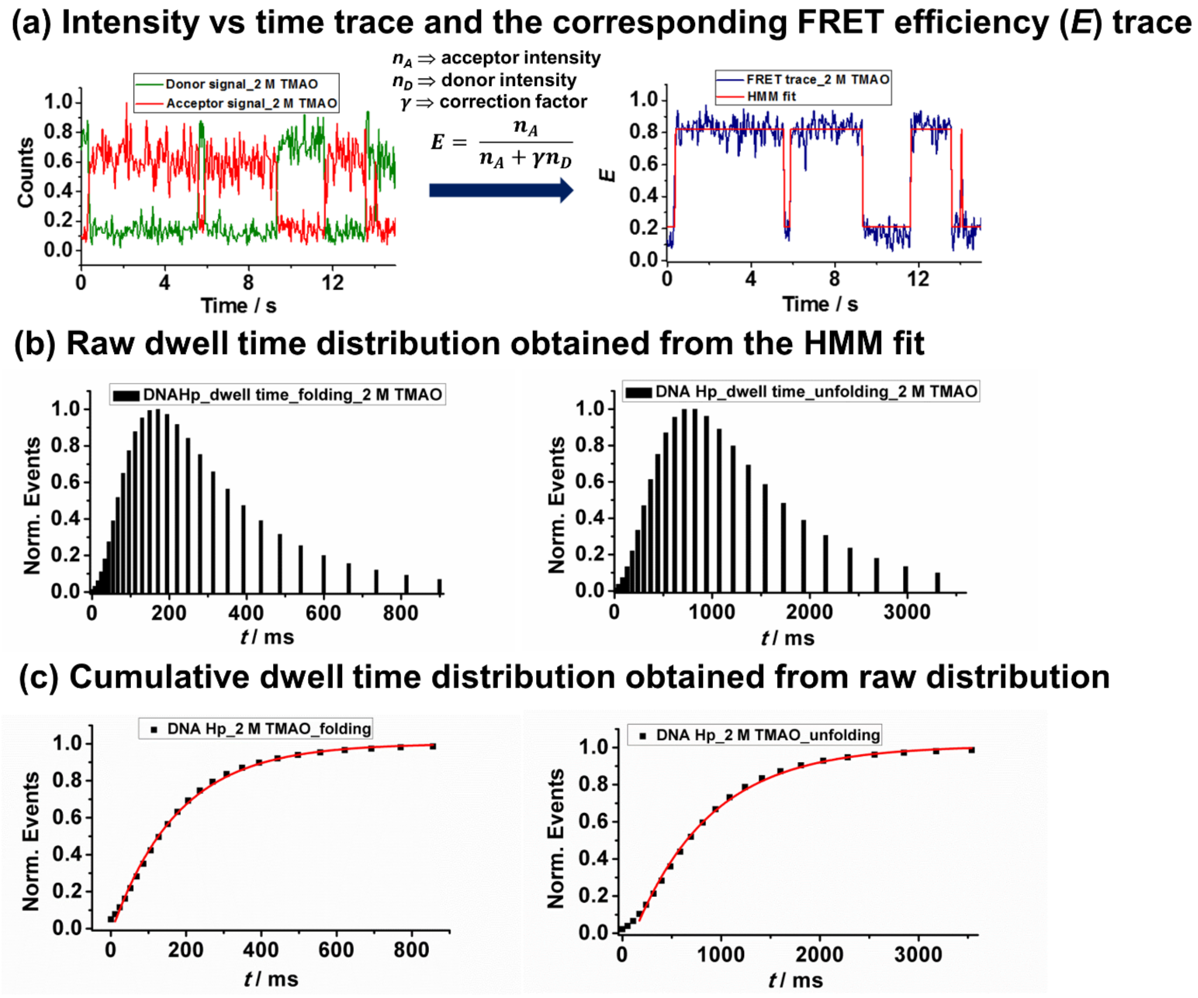

Figure S7. (a) Typical fluorescence traces of the donor acceptor fluorophore labelled DNA Hp in 2 M TMAO and the corresponding FRET time series. The FRET time series shows conformational fluctuations between two states, with $E \approx 0.2$ and $E \approx 0.8$, respectively. The FRET time series are fitted to a Hidden Markov Model (HMM) to obtain the time it spent in each FRET state before making a transition to another FRET state, which is denoted as dwell time. (b) Raw dwell time distribution obtained from the HMM fit to the FRET time series plotted as a histogram where each bar represents the number of events that have the corresponding dwell times. The dwell time distribution plot is obtained from at least 30 such FRET time series obtained from 30 different molecules. (c) Integrating the raw dwell time histograms lead to a cumulative distribution plot, where each point represents the counted events that have a dwell time equal or less than the specified time. These cumulative dwell time distribution plots are fitted to a single exponential function to deduce the dwell time for folding and unfolding, respectively. The dwell time distribution analysis for the open (unfolded) state provides the time scale for folding, while the dwell time distribution analysis of the closed state provides the transition time for unfolding.

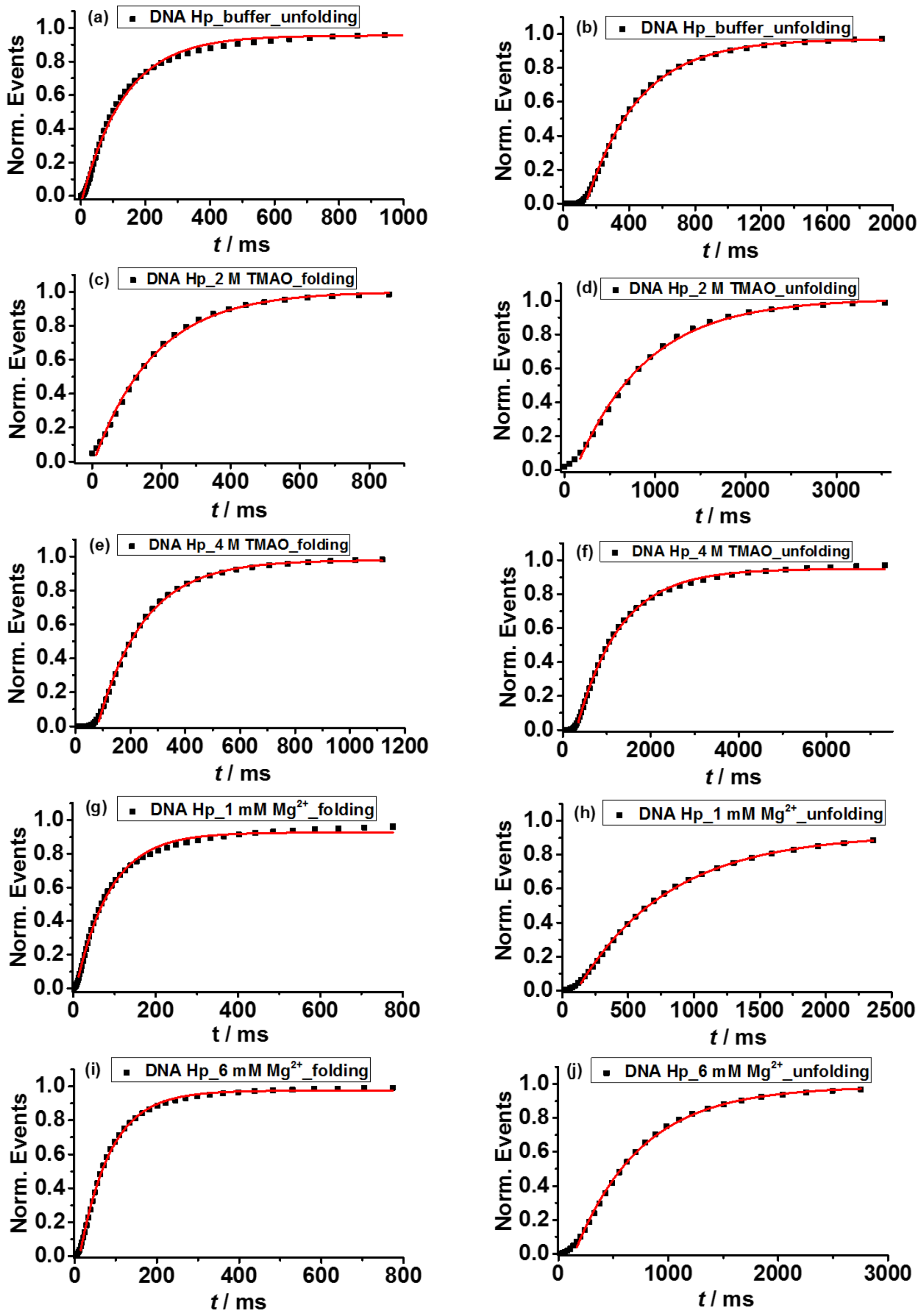

Figure S8. Cumulative dwell time (t) distribution plot in neat buffer and in the presence of different concentrations of TMAO and Mg^{2+}. The raw dwell time distribution histograms obtained from the HMM fit to the FRET time series are integrated to generate cumulative plots where each point indicates the number of counted events that have the dwell time less than or equal to the specified time. These cumulative distribution plots are fitted with single exponential functions (red curve) to obtain the conformational dynamics for that particular transition. The dwell time distribution plot in the open state provides the folding transition time scale while the dwell time in the unfolded state provides the time scale for unfolding.

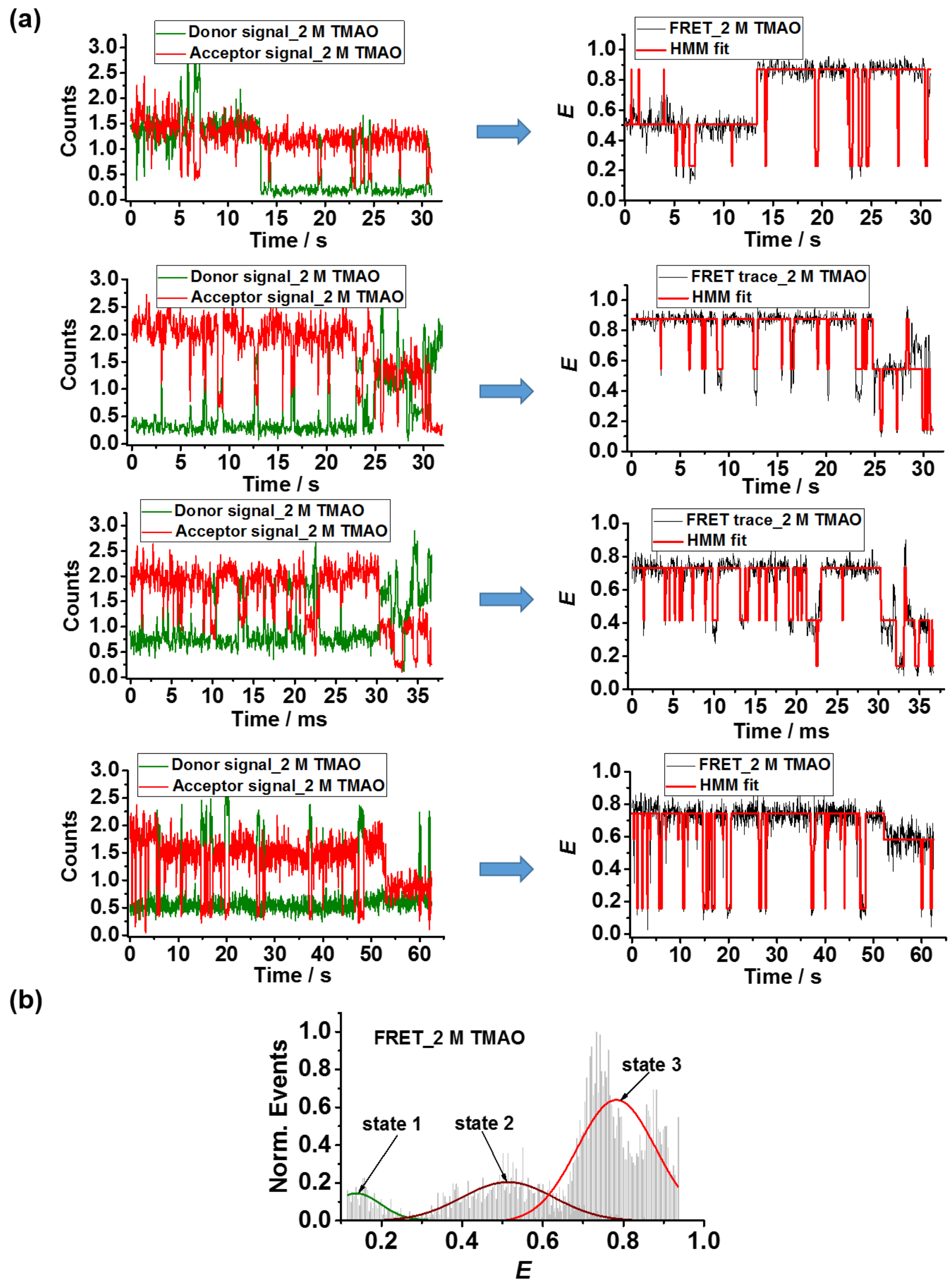

Figure S9. (a) Representative single-molecule fluorescence traces of the Atto 550 and Atto 647N labelled DNA Hp in 2 M TMAO exhibiting a three-state behavior. Corresponding FRET time series are determined from the donor and acceptor fluorescence traces using the *E* = acceptor intensity/(donor intensity + acceptor intensity) data. All the *E* vs. time traces are fitted to a Hidden Markov Model (HMM) to obtain the dwell time, i.e. the time the DNA Hp spent in each FRET state before making a transition to another FRET state. (b) FRET efficiency (*E*) histograms obtained from all these FRET time series showing multiple peaks centred at *E*-values of 0.14 ± 0.01, 0.51 ± 0.01 and 0.78 ± 0.01.

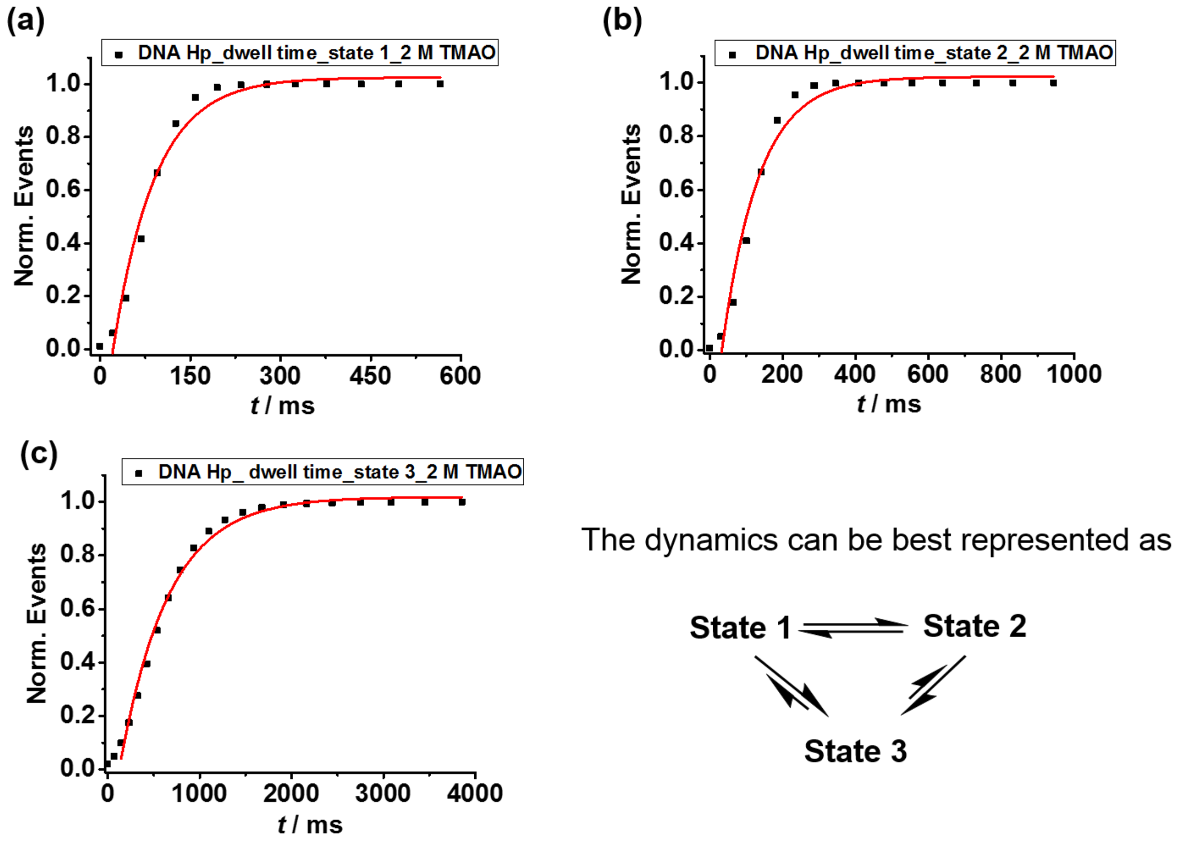

Figure S10. Cumulative dwell time (t) distribution plot obtained for the 10% population of the DNA Hp in 2 M TMAO from the HMM fit to the FRET time series as shown in Figure S7. The FRET efficiency (E) values of state 1, state 2 and state 3 are 0.14, 0.51 and 0.78, respectively. State 1 and state 3 represent fully closed or fully open conformations, respectively, while state 2 denotes partially closed states. The dwell time of state 1 represents the rate for the transition from the fully open state to the fully closed or partially closed state. On the other hand, the dwell time of state 3 represents the dynamics from the fully closed state to the fully open and partially closed state. The rate constant for the transition from the partially closed state to the fully open or fully closed state is obtained from the dwell time of state 3.

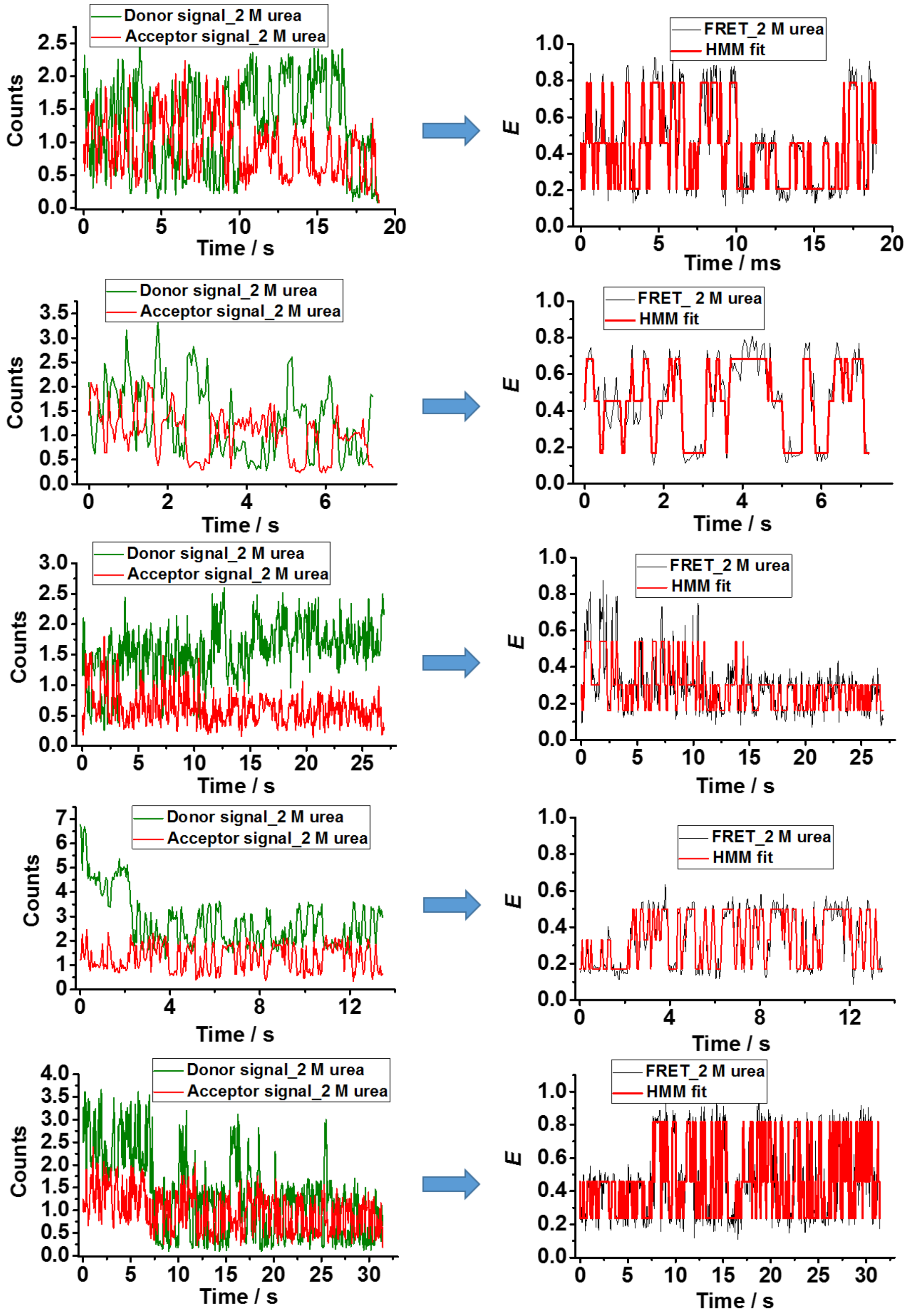

Figure S11. Example of the donor acceptor fluorescence traces of the dually labelled DNA Hp and the corresponding FRET time series obtained in case of 2 M urea. HMM fits reveal a three-state behavior, indicating a rugged free energy landscape.

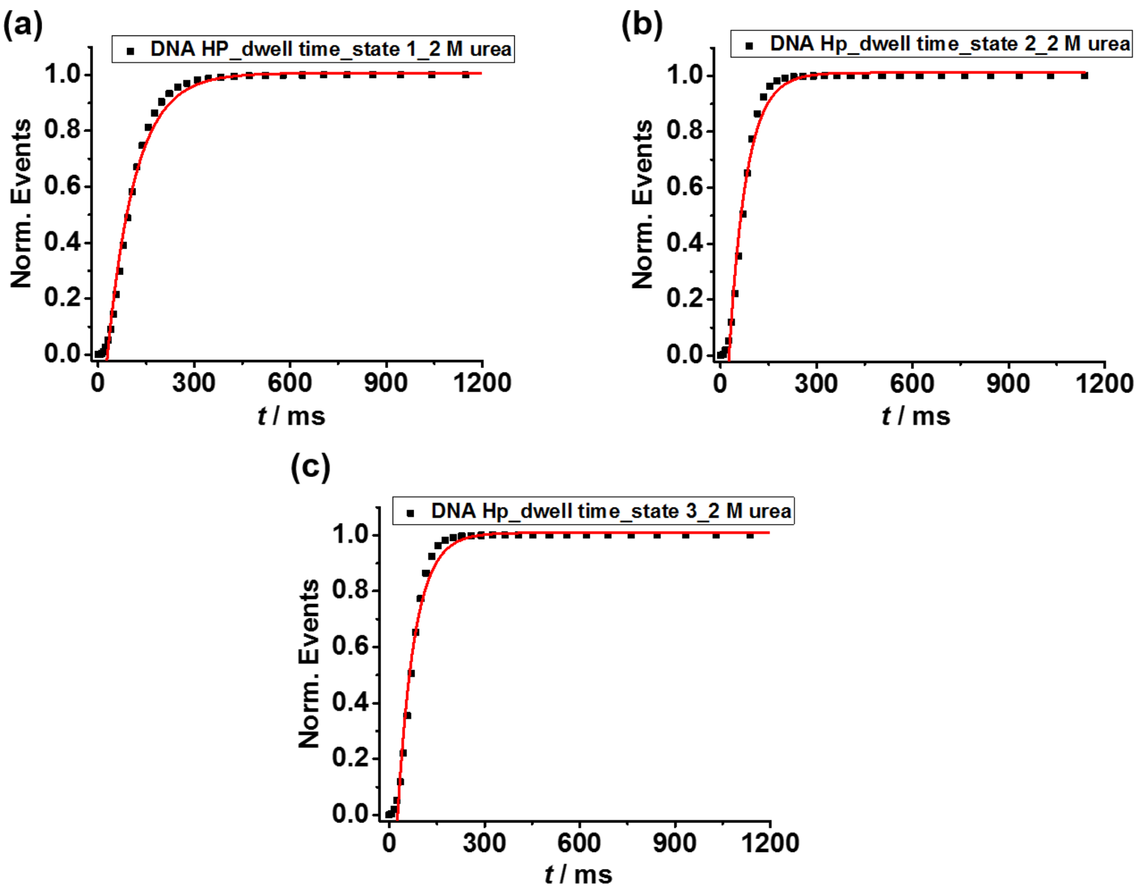

Figure S12. Cumulative dwell time distribution plot for the three states found from the HMM fit to the FRET time series obtained for 2 M urea. The dwell time is determined from the single-exponential fit (red curve) to the dwell time distribution plot. The dwell times obtained for state 1, state 2, and state 3 are 87.1 ms, 54.7 ms and 54.3 ms, respectively.

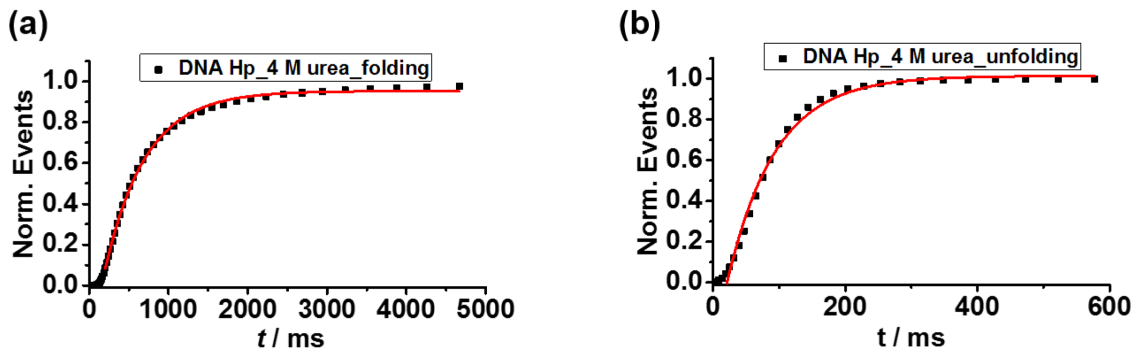

Figure S13. Cumulative dwell time distribution plot for (a) folding and (b) unfolding of the DNA Hp in 4 M urea solution. The dwell time is determined from the single-exponential fit (red curve) to these data. The dwell times obtained for folding and unfolding amount to 515.3 ms and 68.8 ms, respectively.

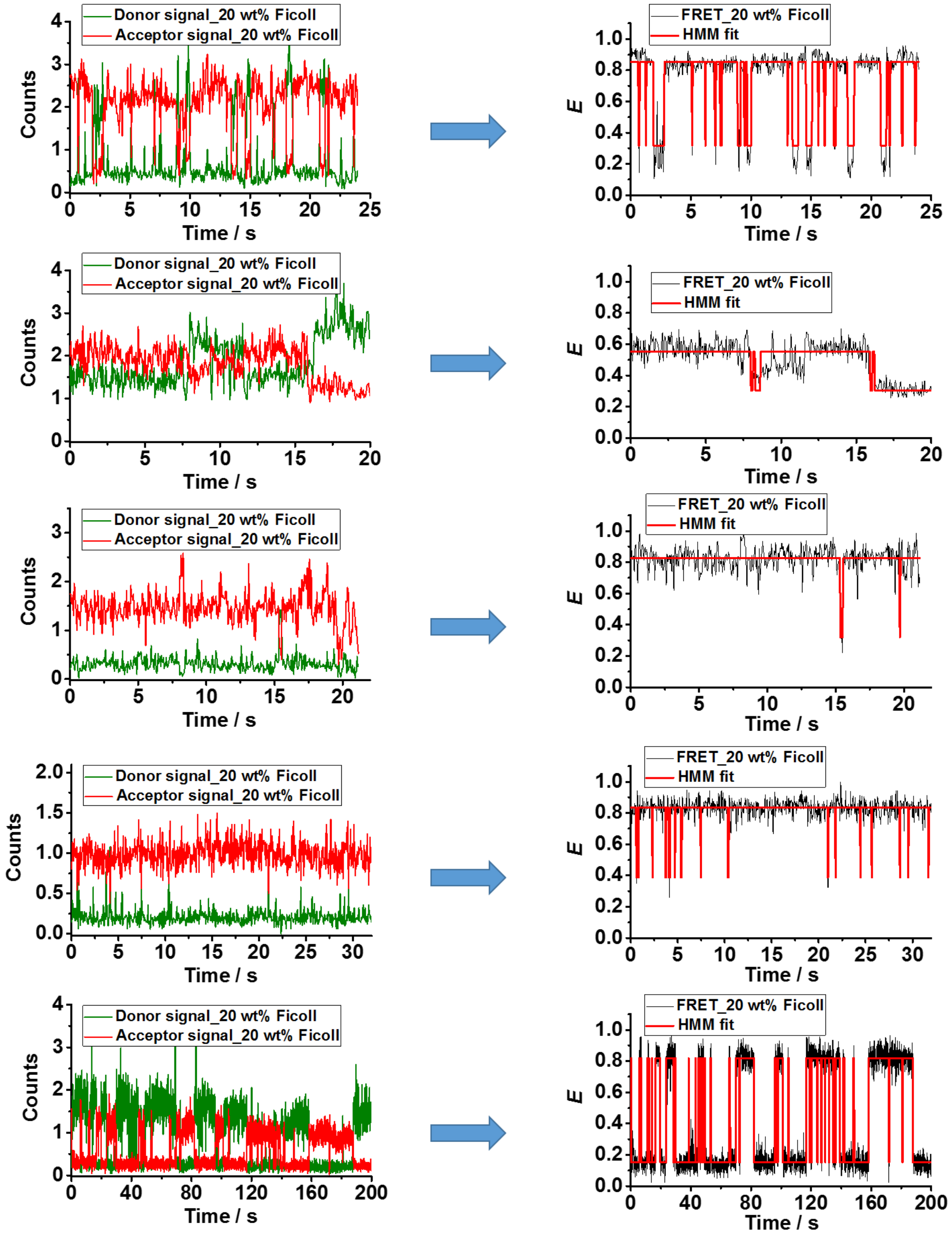

Figure S14. Examples of single-molecule fluorescence traces of the DNA Hp in the presence of 20 wt% Ficoll exhibiting a two-state behavior. The FRET time series were generated from the donor-acceptor fluorescence traces. HMM fits to the time series provided the dwell time distribution plot for folding and unfolding as shown in Figure 6 (main text).
